# Analysing malaria events from 1840 to 2020: the narrative told through postage stamps

**DOI:** 10.1186/s12936-021-03932-7

**Published:** 2021-10-12

**Authors:** Bernard Brabin

**Affiliations:** 1 Clinical Division, Liverpool School of Tropical Medicine, Institute of Infection and Global Health, University of Liverpool, Liverpool, UK; 2 Global Child Health Group, Academic Medical Centre, University of Amsterdam, Amsterdam, The Netherlands

**Keywords:** Malaria, History, Postage stamps, Global eradication campaign

## Abstract

The role played by postage stamps in the history of malaria control and eradication has largely gone unrecognized. Scientific investigators of malaria, especially Nobel laureates, were commemorated with special issues, but the work of the World Health Organization (WHO), which promoted an ambitious and global philatelic initiative in 1962 to support global eradication, is generally overlooked. This review examines the philatelic programme that helped to generate international commitment to the goal of malaria eradication in 1962 and established philatelic malaria icons that had worldwide recognition. Malaria-related postage stamps have continued to be issued since then, but the initial failure of malaria eradication and the changing goals of each new malaria programme, inevitably diluted their role. After the first Global Malaria Eradication Campaign was discontinued in 1969, few Nations released philatelic issues. Since the Spirit of Dakar Call for Action in 1996 a resurgence of postage stamp releases has occurred, largely tracking global malaria control initiatives introduced between 1996 and 2020. These releases were not co-ordinated by the WHO as before, were more commercialized and targeted stamp collectors, especially with attractive miniature sheets, often produced by photomontage. Having a different purpose, they demonstrated a much wider diversity in symbolism than the earlier stylized issues and at times, have been scientifically inaccurate. Nonetheless postage stamps greatly helped to communicate the importance of malaria control programmes to a wide audience and to some extent, have supported preventive health messages.

## Background

Postage stamps are often issued to commemorate special events or noteworthy individuals. They may promote new public initiatives and even political agendas [1, 2]. Stamps have played a role in health-related government campaigns, such as anti-smoking, disability support, blood donation, cancer awareness, surgery interventions, commemoration of World Health Days, HIV awareness, and currently COVID control [3–10]. Stamps celebrate World Health Organization (WHO) anniversaries and pay tribute to medical, individual, or scientific discoveries. Not confined to one country, the same themes recur, highlighting the international nature of health delivery, and the many scientific contributions to important breakthroughs [11, 12]. Stamps remain an additional route for broad diffusion of information, even though letter writing has declined. Messages aside, they may provide a route for raising health funds - a good example of which is their use for anti-tuberculosis activities. First employed in 1897, a unique idea was created of “charity’ stamps - in this case marking both Queen Victoria’s Diamond Jubilee and raising money for Queen Victoria’s Homes for Consumptives Fund [13]. Adding surcharges to non-commemorative stamps raised funds for tuberculosis control throughout the 20th century [14].

The allure of postage stamps and postmarks has become a commercial and pictorial means to engage attention, with mail being passed from hand to hand, domestically and internationally, reaching even remote areas. By use of symbols, slogans, and pictures, a message can be dispersed far and wide. Despite its small size and discrete purpose (letter or parcel), the stamp is postulated to capture a more concentrated health message density per sq. cm than any other cultural form [15]. Others have referred to stamps as ‘icons’, meaning that, like icons in art more generally, they have the ability to capture depictions and symbols that speak to different cultures about the same health event, although the impact of such messages through postage stamps would be almost impossible to measure.

Malaria postage stamps are prolific. A descriptive presentation volume on parasitological topics, which included a pictorial listing of malaria postal issues, was published in 1981 to commemorate the 80th birthday of Professor P.C.C. Garnham [16]. Subsequently a descriptive philatelic outline of parasites on stamps, which included malaria themes, was published [17, 18]. These collections do not reveal the extent to which the malaria themes they encapsulated had changed over time, or how they related to international malaria control initiatives. Since 1996, malaria control strategies were revised in line with the lessons learnt from the successes and failures, and postage stamps tracked this progress and proved useful in broadcasting new messages. This paper aimed to document and critically examine the messages and stamp designs that accompanied and were used to broadcast to the public the changing initiatives to investigate, control and/or eradicate malaria up to the present time.

## Methods

### Search strategy

All countries included in the most recent six volume Stanley Gibbons Stamps of the World (1840–2014) catalogue were screened for stamps directly related to malaria [19]. Specific annual catalogues published by Yvert and Tellier were reviewed [20]. Catalogues had listings of stamps from verified national postal agencies (i.e., stamps issues for use on mail, not as collector’s items) since 1840. Excluded from this review were unofficial ‘special’ stamps produced by private organizations, which require additional Government postage for mail transmission, and are not catalogue listed. It was noted that for 20th century issues up to 2007, Borgsteede had identified 539 malaria-related stamps, and 567 with a general theme related to protozoa, including malaria [17]. On-line listings of national postal administrations were checked to March 2021 using Web search engines. PubMed.gov was searched for references in English or French using the terms malaria, philately, stamp, postage stamp, paludisme, timbre, quinine, cinchona, Laveran, Ross. Private collections, including the Prout reference stamp collection at the Wellcome Trust Library, were consulted [21].

### Analytic approach

Five historical periods were considered: the 19th century with a focus on investigators’ contributions to malaria research; the 20th century pre-1955 and prior to the commencement of the first WHO Global Malaria Eradication Campaign; 1955–1969, the period of the first Global Malaria Eradication Campaign [22]; 1970–1996, covering discontinuation of this campaign up to the date of the Spirit of Dakar Call for Action [23, 24]; and 1997–2020, the period following the Spirit of Dakar Call for Action to the present [25]. Analysis of stamps designs was informed by semiology, such that postage stamps were examined as unique signs, with the capacity to convey messages in a confined space. The country and date of issue, face value, overprints or surcharges were assessed, as were the variety of symbols, images, slogans, recurring themes, national campaigns, historical scenes and personalities, expressions of solidarity, and colours used. Examples used for illustration were selected on the grounds of clarity, technical innovation, or simplicity of expression, or based on the First Day Cover envelope for a selected postage stamp.

### Terminology

*Commemorative* refers to a stamp designed to commemorate an event or personality. The term is often used loosely to cover all postage stamps issued for special occasions for a limited period, and for a specific theme. *Overprint* refers to an inscription added to the stamp design after the basic stamp has been printed and does not alter the value of the stamp. A *miniature sheet* is a single or small group of postage stamps still attached to the sheet on which they were printed. The margins of the sheet may have ornamental designs, emblems and logo(s) which are not the part of stamp(s). *Icon* is defined as a pictorial sign; and *symbol* as a conventional sign-for example a picture, letters, or numbers. A symbol can become an icon when it receives a noticeable degree of typographical definition or is placed in a prominent and isolated position [15].

### Historical discoveries

**Fig. 1:**
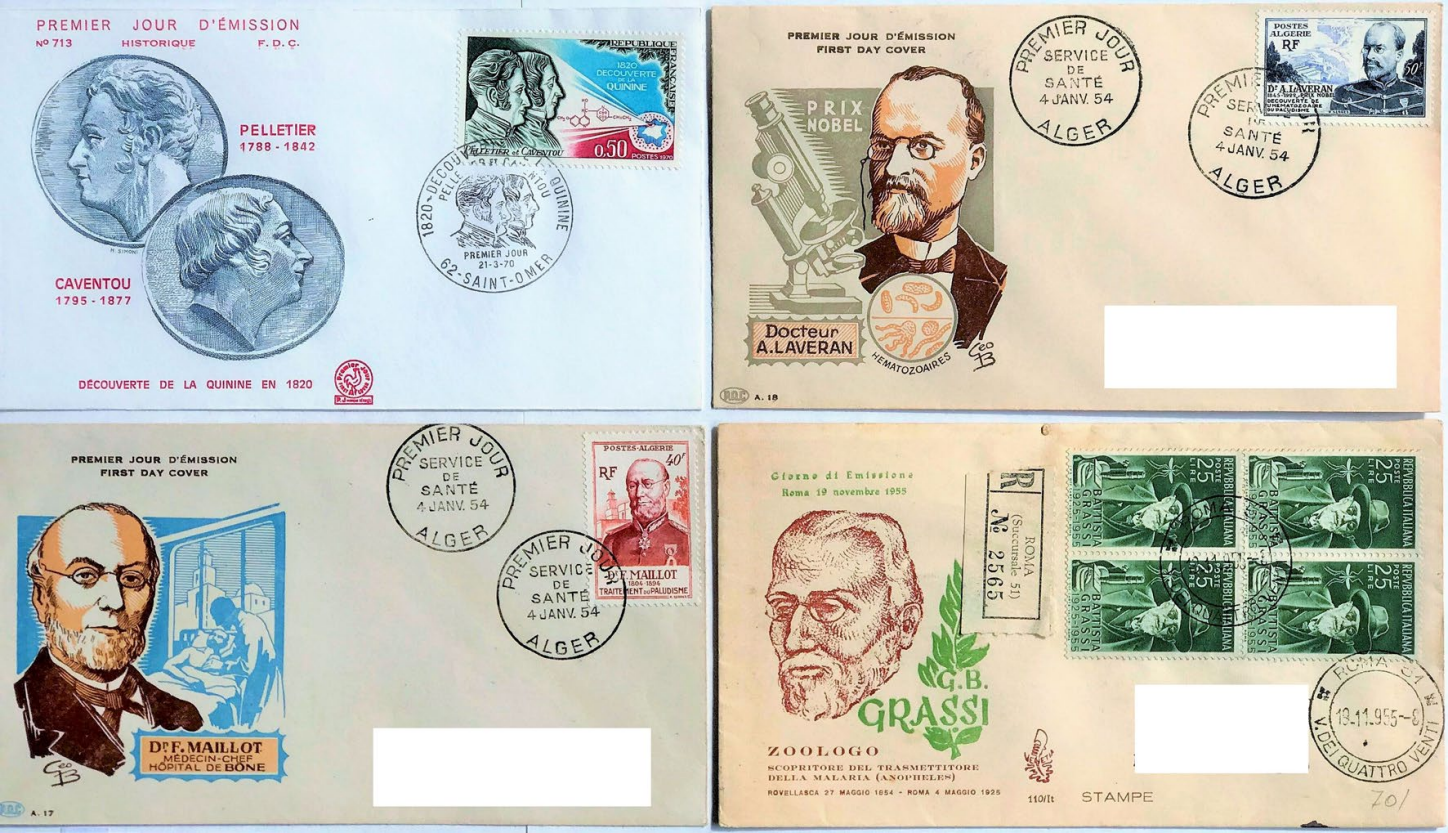
First day Covers: Pelletier and Caventou (1970), Maillot (1954), Laveran (1954), and Grassi (1955). Brackets: year of issue

**Fig. 2:**
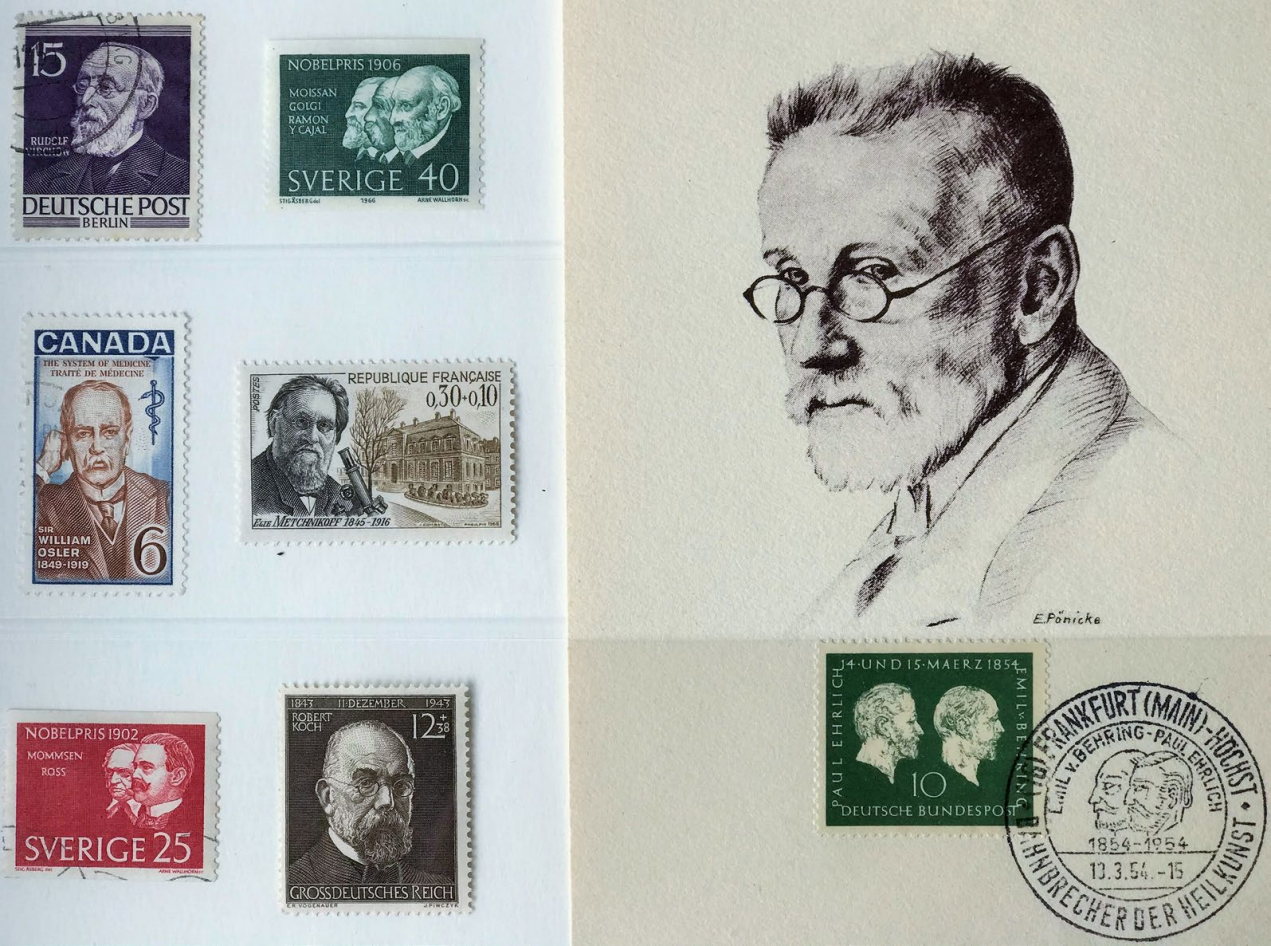
Postal issues of 19th century malaria investigators. Brackets: year of issue. Top left, to bottom right: Rudolf Virchow (1944), Camillo Golgi (1966), William Osler (1969), Élie Metchnikoff (1966), Ronald Ross (1962), Robert Koch (1944), Paul Erhlich (1954), portrait and stamp on the right. Ross (Physiology and Medicine) is shown with co-Laureate Monmmsen (Literature), and Golgi with Ramon y Cajal (joint prize in Physiology and Medicine) and Moissan (Chemistry)

**Fig. 3:**
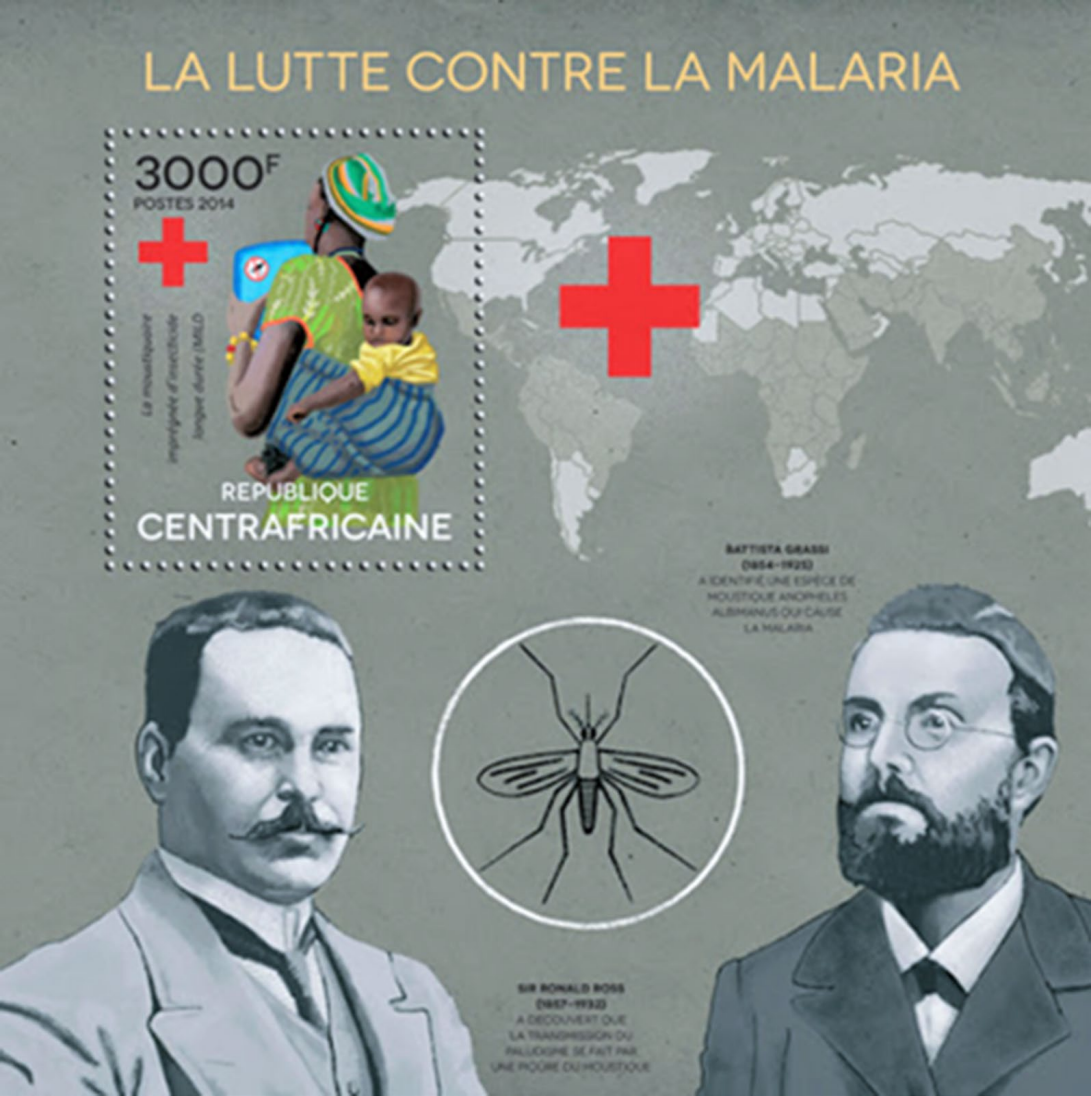
Central African Republic miniature sheet showing Ross and Grassi. Issued in 2015

Postage stamps contain historic images of individuals and national identifiers that are archives of both scientific and international heritage [26]. They form an important visual record of the history of medicine and its commemoration. Table 1 summarizes 19th century contributions of twenty investigators who made significant advances in malaria knowledge, twelve of whom were philatelic subjects [27–45]. Maillot and Laveran, who were both serving officers in the French army in Algeria, were represented within a set commemorating pioneers of military health services (Fig. 1). Laveran became a national hero for his discovery of the parasitic cause of malaria and was portrayed on several later stamps commemorating Nobel laureates. Postage stamps were issued showing Golgi, Metchnikoff, Erhlich, Ross and Koch, all Nobel laureates, as illustrated in the first philatelic issues of some of these investigators shown in Fig. 2. Despite their major contributions and national recognition, Nobel laureates were not awarded to Virchow, Osler, Grassi, or Manson, who Grassi considered first formulated the mosquito theory [46], and alerted Ross to the solution of this hypothesis [47]. Ross himself preferred to emphasize Laveran’s, rather than Manson’s views on the hypothesis and Grassi eventually became an adversary of Ross. A strong sense of competition between German bacteriologists and Italian malariologists followed Robert Koch’s 1898 visit to Italy, which produced further interest in the mosquito theory [48]. Koch was appointed as a ‘neutral arbitrator’ for the 1902 Nobel committee and threw the full weight of his considerable authority in insisting that Grassi did not deserve the honour. The argument on the Italian contribution to the discovery of the malaria transmission cycle still rumbles on [48]. A recent 2015 postage stamp from the Central African Republic shows Ross separated by a mosquito from Grassi, suggesting equivalence in their contribution (Fig. 3). Koch became a major national and international icon, since celebrated on more than forty commemorative stamps from different countries, numerically exceeding those honouring even Louis Pasteur. Less famous, Dr. Julius Wagner-Juaregg (1857–1940), who was awarded the Nobel Prize in 1927, was shown in an Austrian stamp in 1957 [49]. He had successfully used inoculations of infected blood from patients with tertian malaria as fever therapy for treatment of syphilitic dementia paralytica.

**Table 1:** Nineteenth century investigators' contributions to malaria research and commemorations by national postal services

Investigator	Year of event	Nobel Prize (year)	Contribution to malaria research	Postal Issue	Year of issue	References
Pierre Pelletier (1788–1842) & Joseph Caventou (1795–1877)	1820	–	Isolated quinine from bark of Peruvian trees of genus *Cinchona*; clinically tested the product and set up manufacturing facilities	Yes^a^	France 1970	[27]
François Maillot (1804–1894)	1836	–	Brain at autopsy coloured grey with many tiny dark areas. Successful use of quinine sulphate for febrile Algerian army staff (1834)	Yes^a^	Algeria 1954	[28]
Heinrich Meckel von Hemsback (1790–1829)	1847	–	Recognized pigmented bodies among red cells and in the organs in a patient who died of malaria	No	–	[29]
Rudolf Karl Virchow (1821–1902)	1848	No	With Friedrich Frerichs (1919 –1885) associated the black pigment specifically with malaria	Yes	Germany 1948	[30]
Louis Achille Kelsch (1841–1911)	1875	–	Concluded malaria diagnosed by presence of melanin, which was found in blood cells, liver, spleen, and marrow	No	–	[31]
Charles Alphonse Laveran (1845–1922)	1880	Yes (1907)	Announced to the French Academy of Medicine the discovery of the living malaria parasite	Yes^a^	Algeria 1954	[32]
Ettore Marchiafava (1847–1935)	1885	No	With Angelo Celli (1857–1914) showed parasites inoculated from man to man and that pigmented granules were external to leukocytes	No	–	[33]
Camillo Golgi (1843–1926)	1885	Yes (1906)	Described development of segmenting forms in blood of quartan parasites; identified association with cyclical fevers and difference between quartan and tertian fevers	Yes	Sweden 1966	[34]
William Councilman (1854–1933)	1885	No	Identified with George Sternberg (1838–1915) red cell hyaline-like bodies similar to Laveran’s and causal of malarial fever	No	–	[35]
William Osler (1849–1919)	1886	No	Confirmed presence of parasites patients in USA; showed diagnostic value of malaria blood slide in all fevers	Yes	Canada 1969	[36]
William Welch (1850–1934)	1886	No	Marchiafava and Celli name the parasite *plasmodium* and Welch *P.falciparum* as recognized species formed crescents	No	–	[37]
Élie Metchnikoff (1845–1916)	1886	Yes (1908)	Showed the relation of the parasite to the sporozoa. Developed eosin and methylene blue slide preparations	Yes	France 1966	[38]
Vasili Danilewsky (1852–1939)	1886	No	Recognized similarity of avian haemosporidia and human parasites; described exflagellation of avian gametocytes (1889)	No	–	[39]
Paul Erhlich (1854–1915)	1891	Yes (1908)	First to successfully treat malaria using methylene blue	Yes	Germany 1954	[40]
Patrick Manson (1844–1922)	1894	No	Proposed flagellating bodies in mosquito stomach developed in water when mosquito dies; stated flagellation was extracorporeal phase and proposed transmission by a ‘suctorial insect’; in 1900 showed subjects in London were infected by mosquitoes sent from Italy	No	–	[41]
William MacCallum (1874–1944)	1897	No	Observed fertilization of crescent and flagellate forms in avian malaria identifying its sexual reproduction	No	–	[42]
Ronald Ross (1857–1932)	1898	Yes (1902)	Showed that *Proteosoma*, a malaria parasite of birds, was conveyed by mosquitoes	Yes	Sweden 1962	[43]
Giovanni Grassi (1854–1925)	1898	No	With Amico Bignami (1862–1929) and Giuseppe Bastianelli (1862–1959) succeeded in infecting man by mosquitos from malarial regions; suggested preliminary tissue phase existed; showed malarial parasites carried by female *Anopheles*	Yes^a^	Italy 1955	[44]
Robert Koch (1843–1910)	1899	Yes (1905)	Initially disbelieved Laveran’s findings. With Kossel identified parasites in lower monkeys (named *P. Kochi* by Laveran). First to develop ‘carrier’ hypothesis in asymptomatic individuals, and eradication strategy with quinine prophylaxis and surveillance	Yes	Germany 1944	[45]

^a^ Selected postage stamps shown in Fig. 1

**Fig. 4:**
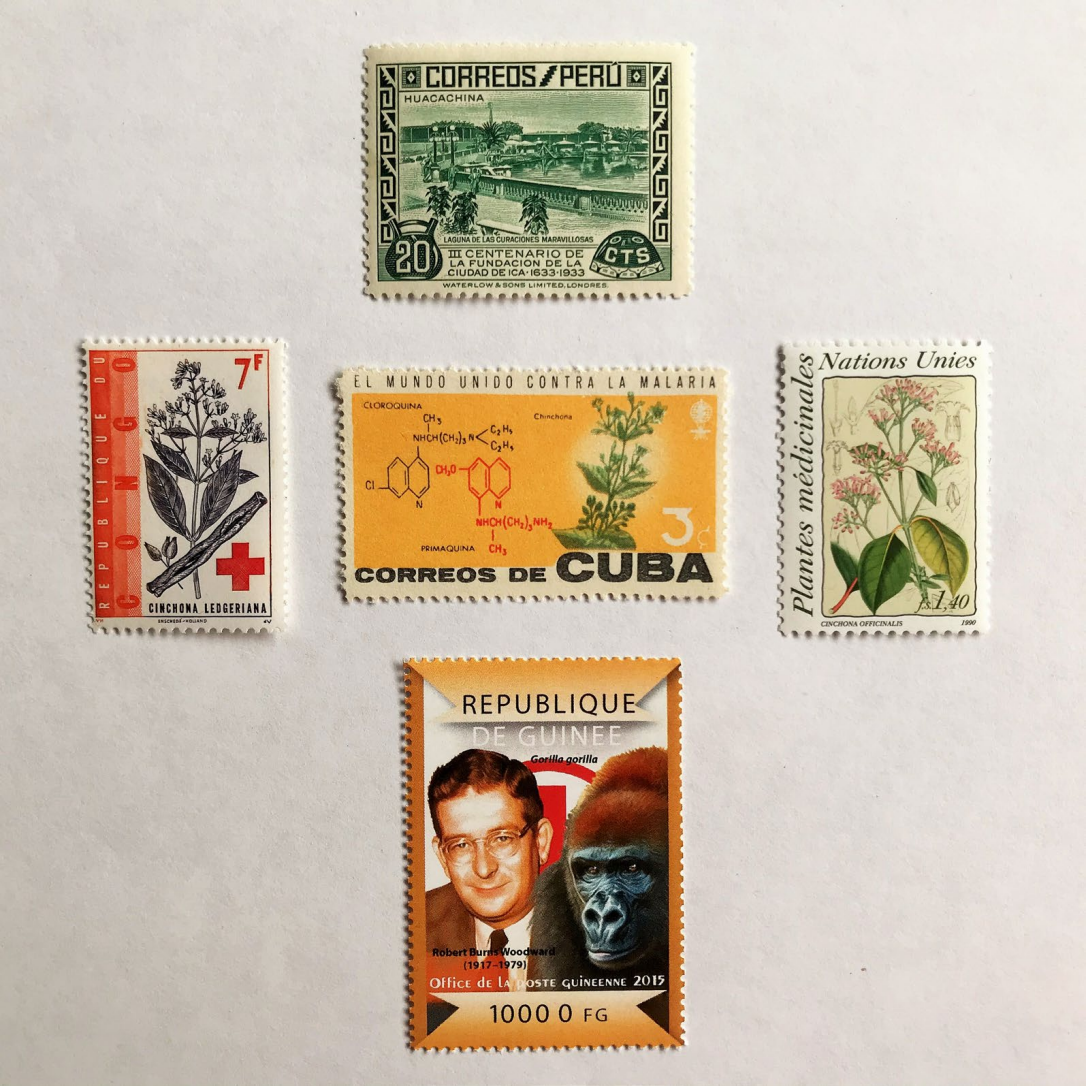
Postage stamps, the Cinchona plant and quinine synthesis. Issue year: Peru (1935) Miraculous lake water which cured fevers; Cuba (1962); Republic of Congo (1962); United Nations (1990). République de Guinée commemorative issue (2015) showing Bob Woodward, who first synthesized quinine

Arguably the effects of quinine were first discovered when an earthquake caused cinchona trees to fall into a Peruvian lake (the Lagunade De Las Curaciones Maravillosas) near Loxa and whose waters cured febrile illness. The lake was credited with miraculous healing [50]. In 1820 in Paris, the toxicologist Pierre Joseph Pelletier (1788–1842) and a pharmacist Joseph Caventou (1795–1877) successfully isolated the quinine alkaloids, demonstrating their efficacy in intermittent fevers (Fig. 1) [27]. In 1935 the Peruvian postal administration issued two stamps showing the ‘miraculous’ lake in a set commemorating the tercentenary of quinine’s arboreal origins (Fig. 4). Charles Marie de La Condamine (1701–1774), the celebrated French explorer, geographer and mathematician who unsuccessfully endeavoured to bring Cinchona trees and seeds from Ecuador to France [51], was recognized in a 250th year commemoration stamp (1993) from Ecuador showing the tree with its flowers (*Cinchona cordifolia*). Various cinchona plant species have featured on stamps issued from the Republic of Congo (1962) (*Cinchona ledgeriana*), Colombia (1983) (*Cinchona lancerfolia*), The United Nations (1990) (*Cinchona officinalis*), Peru (2017) (*Cinchona pubescens*), and with species unspecified from Poland (1962), Cuba (1962), Senegal (1969) and Rwanda (1970) (Fig. 4; see Fig. 8 for Polish issue). It was not until 1944 that the Nobel Laureate in chemistry Robert Burns Woodward (1917–1979) first synthesized quinine, commemorated in a stamp from the République de Guinée (2015) (Fig. 4). It is unclear why emphasis is given to a non-human primate in this stamp image, which provides a confusing message.

### From 1932 to 1954 prior to first global malaria eradication campaign

Mosquito control measures proved successful in the American South in the 1910s. New approaches to malaria control in the first half of the 20th century were driven by The International Health Board (1913–1928) and the Rockefeller Foundation (1913-present). World War One further demonstrated the risks of epidemic malaria [52], which was treated with quinine [53]. The evolving knowledge on malaria was accompanied by malaria-related stamps utilizing an extraordinary variety and range of themes, formats, and design characteristics.

**Fig. 5:**
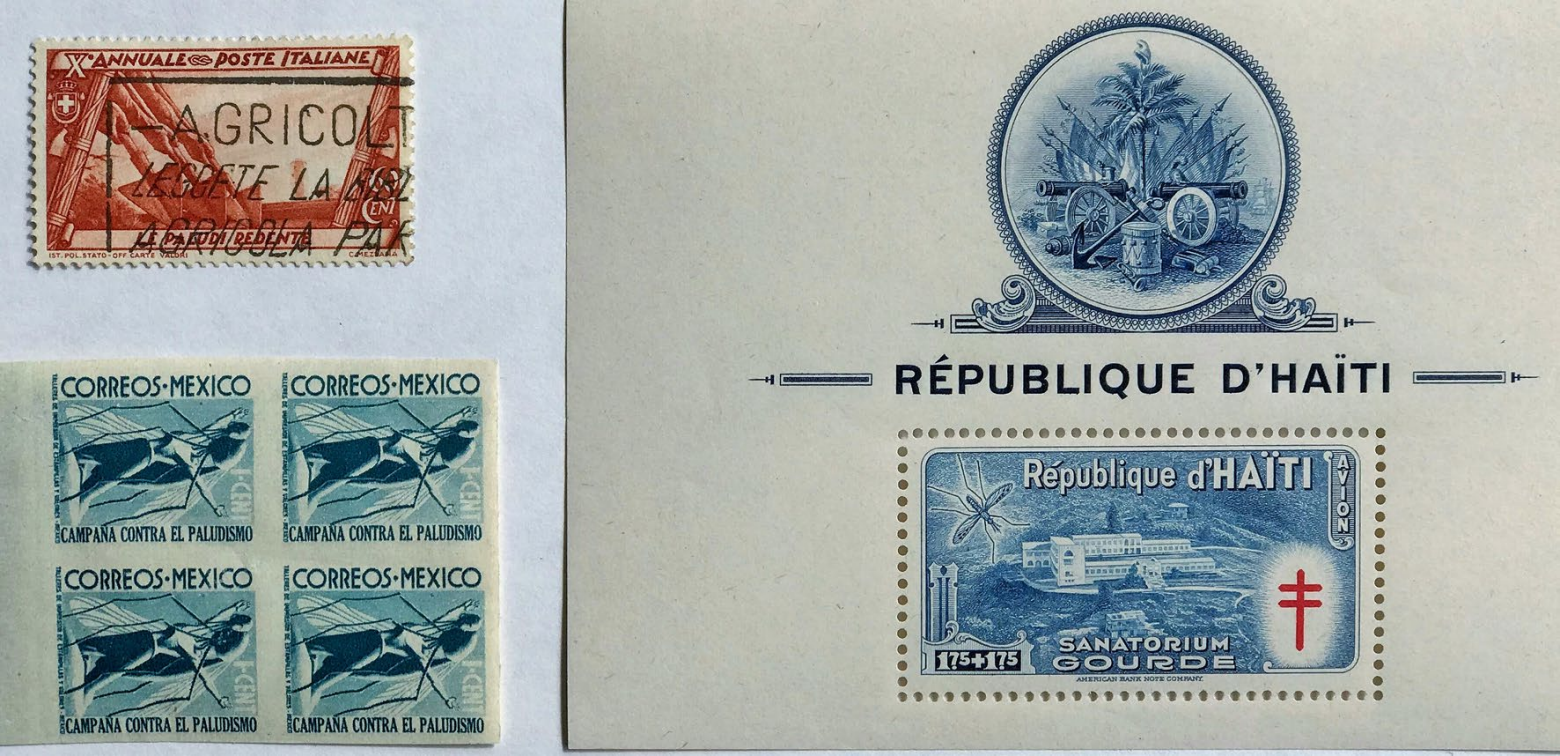
1932–1955 stamp issues related to malaria. Issue year: Italy (1932); Mexico (1935); Haiti (1949) issued in seven values

The first postage stamp to promote a malaria control strategy was issued in 1932 by the Italian Government, although it formed only one 60 cent stamp within a large anniversary series to commemorate the 10th anniversary of the March on Rome by the Fascists in 1922. It showed three hand spades and was inscribed ‘paludi redenti’ (marsh reclamation) (Fig. 5). The stamp was designed to demonstrate that land reclamation was required to reduce marshland and mosquito breeding and improve land use [53]. In 1939, the Mexican government issued a blue, one centavo stamp, which was obligatory on all mail. Effectively this was a charity stamp surcharge to facilitate malaria control activities. The stamp portrays a kneeling man with a monstrous mosquito on his back, symbolizing the heavy malaria burden, and it was re-issued in 1944 and 1947 (Fig. 5). This stamp issue and its ‘fiscal tax’ financed 15% of malaria control activities and reflected the Mexican Government’s early commitment to malaria control [54]. From 1942 to 1958 malaria mortality and morbidity in the country went respectively from 129 to 44 and 847 to 21 per 100,000 population, a decrease that largely preceded the Global Eradication Programme in 1962 [54].

Paul Müller (1899–1965) a Swiss chemist, first developed and tested dichlorodiphenyltrichloroethane (DDT) as an insecticide (Nobel prize Physiology and Medicine, 1948) and patented the product in 1940 [55]. His work was commemorated in a stamp in 2015 from the République de Guinée. A clear strategic technical solution emerged in favour of preventive anti-mosquito regimens in preference to curative medical approaches.

The international Rockefeller malaria control programme began in Italy from 1923 to1951, with projects in India from 1936 to 1942, and trials of DDT commencing in Mexico in 1942. The Rockefeller Foundation promoted an insecticide-based global attack on malaria, and by 1943 industrial DDT production had commenced in the USA and Great Britain [53]. This use of DDT, including its strategic role during World War Two, inspired the newly founded WHO (1948) to begin national anti-malaria campaigns. These anti-mosquito campaigns were successful in many low-income countries. Aspirations towards unity in variety, a sharing with the world of national, cultural, and scientific achievements embodied the spirit of the newly founded WHO [15]. The Haitian government in 1949 issued a series of seven postage stamps which embraced these values, the price of which included a surtax for malaria control operations and tuberculosis. They all showed a TB sanitorium along with a mosquito (species not stated), reflecting two infections with high health costs (Fig. 5).

The demonstrable effectiveness of DDT strongly appealed to those who wanted to eradicate malaria and influenced the WHO global anti-malaria eradication programme that began in 1955 [22]. By 1953, the WHO, with UNICEF, had established 35 Malaria Control Demonstration Teams and malaria was eliminated in countries where the mosquito was easily controllable (USA, Italy, Guyana, Greece, large parts of Venezuela) [22]. George Giglioli (1897–1975), who successfully promoted its use in Guyana from 1946, is shown on a later Guyanese stamp issued in 1978 [56].

### The first global eradication campaign from 1955 to 1969

**Fig. 6:**
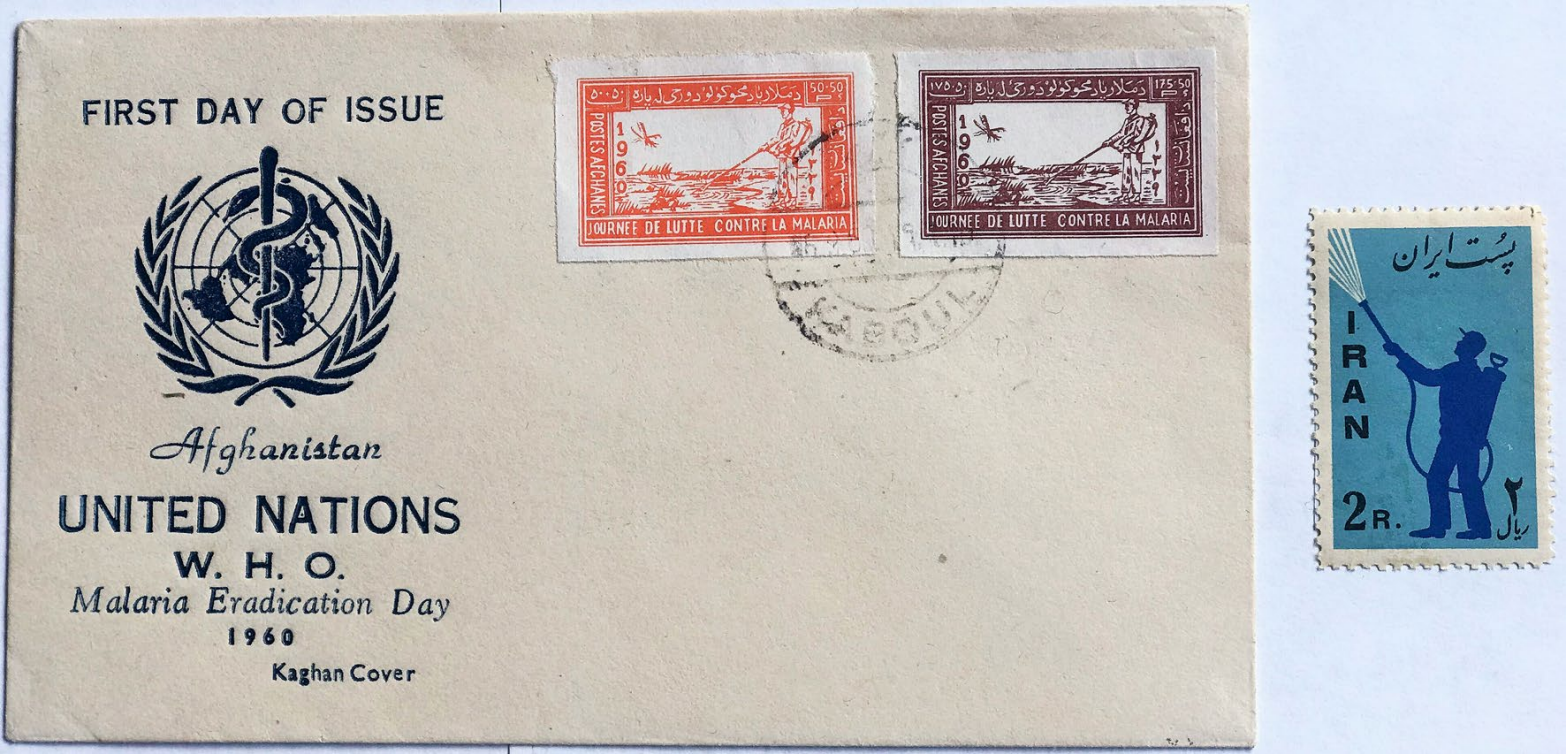
First issues demonstrating insecticide spraying from Afghanistan and Iran. Both issued in 1960

**Fig. 7:**
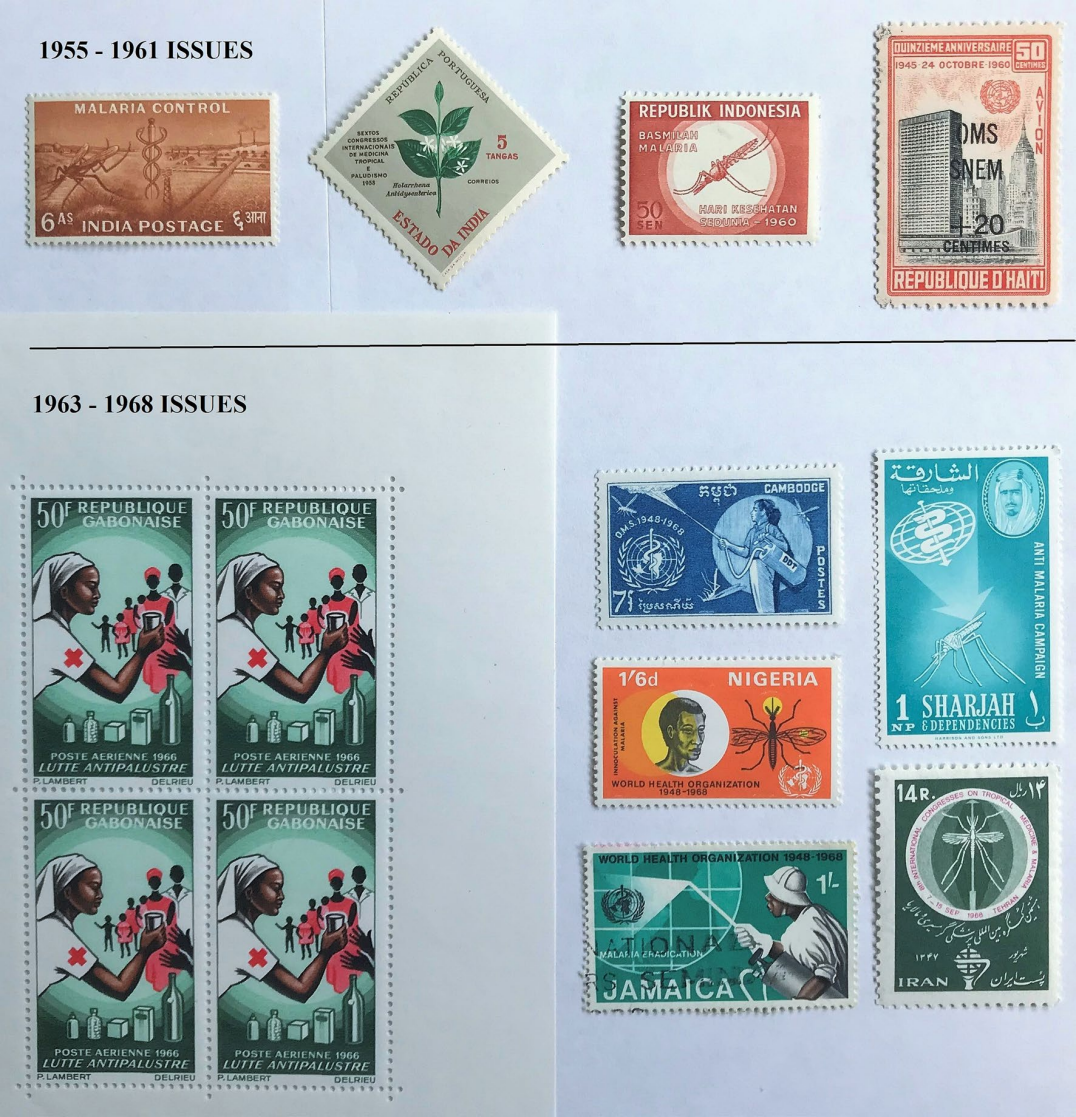
Stamp issues between 1955–1961 and following the 1962 WHO omnibus issues between 1963–1969. Brackets: year of issue. India (1955); Estado da India, 6th International Congress of Tropical Medicine and Malaria (1958); Indonesia (1960); Haiti (1961); Sharjah (1963); Gabon (1966); Jamaica (1967); Iran, Cambodia, and Nigeria (all 1968). The 1961 UNICEF 15th Anniversary issue illustrating insecticide sprayers is not shown

Images of insecticide spraying were first shown on stamps from Afghanistan in 1960 promoting the United Nations Malaria Eradication Day, and on a First Day Cover showing the WHO Logo. In the same year, similar images were used in an Iranian stamp (Fig. 6). Images of residual insecticide spraying were widely used as a theme elsewhere in later issues in the 1960 s as well as in the omnibus issues of the 1962 Global Malaria Eradication Campaign (Fig. 7).

In 1955 the first WHO Global Malaria Eradication campaign was launched as a worldwide effort to eradicate malaria. In that year, the 8th World Health Assembly (WHA) held in Mexico passed a resolution calling for malaria eradication within five years (although it excluded Africa) [57–59]. Early reports from Venezuela, which had the highest number of malaria cases in Latin America before 1935, supported scientific assurances that eradication was feasible and fired the imagination of many distinguished and experienced specialists [60]. Use of the residual insecticide DDT seemed an almost miraculous way of depleting *Anopheles* numbers and longevity [58]. Over the next 20 years an immense effort was deployed by some 20 countries, co-ordinated by the WHO and generously supported by bilateral funds [61].

Postage stamps accompanying this major initiative illustrated the variety of ways in which malaria icons could be adapted, transformed, and promoted. In 1955 the government of India issued a 6 anna stamp that depicted an anopheline mosquito, a caduceus (an ancient Greek or Roman herald’s wand, typically one with two serpents twined round it, carried by the messenger god Hermes or Mercury) and a factory, possibly representing the chemical production of DDT (Fig. 7). Postage stamps proved a popular method to depict the war on malaria. Also, in 1955 Italy issued a stamp to commemorate the 30th anniversary of the death of Giovanni Battista Grassi (1854–1925) (see Table 1), showing his image, an *Anopheles*, and a microscope (Fig. 1). Portugal and its colonies in 1958 issued stamps to commemorate the 6th International Congress of Tropical Medicine and Malaria held in Lisbon (Fig. 7). This was followed in 1960 by Iran, and Indonesia, with four stamps for a National Malaria Eradication Day showing *Anopheles* and the words ‘Destroy Malaria’ and ‘World Health Day’, and Haiti with an air mail surcharge overprint (Fig. 7). In 1961, Turkey also included a single stamp showing insecticide sprayers in a set of three stamps celebrating the 15th Anniversary of UNICEF. Between 1939 and 1961 twelve countries issued 35 postage stamps with a theme either directly or indirectly related to malaria. These became the forerunners for the 1962 malaria eradication stamp issues [62].

**Fig. 8:**
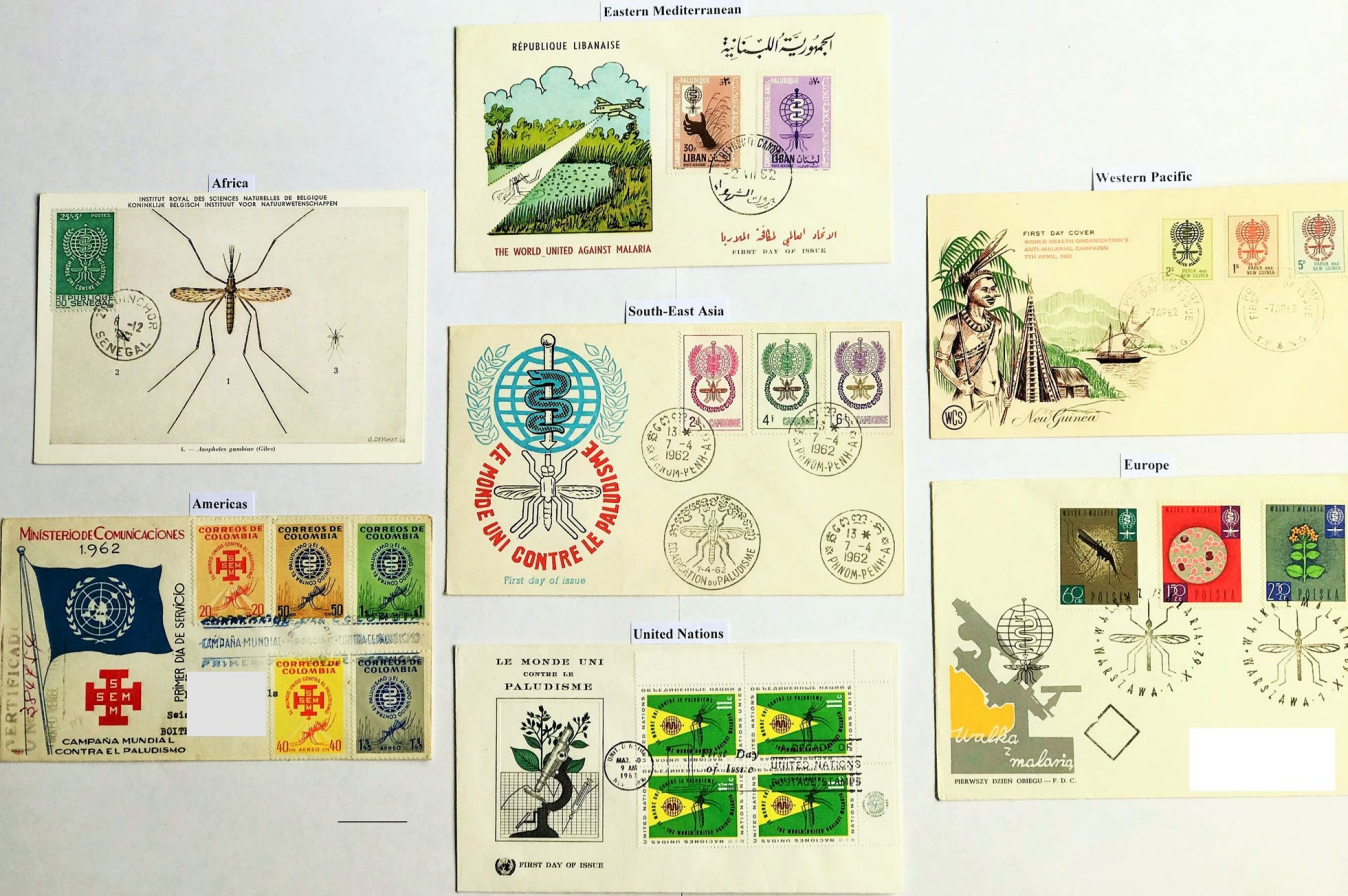
Omnibus issues of 1962 from the six WHO regions and the United Nations. Examples include: Eastern Mediterranean (Lebanon), Western Pacific (Papua New Guinea), Americas (Colombia), South-East Asia (Cambodia), Africa (Senegal). An identical stamp design to that shown for Senegal was used for the following African countries: Central African Republic, Mali, Republic of Congo, Niger, Côte d’Ivoire, Gabon, Comores Islands, Mauritania, French Somalia, Dahomey, Malagasy, French Cameroun, Upper Volta. These African issues were the first malaria-related stamps to be issued by any African Nation. The postcard with the Senegalese stamp is reproduced with permission of the Koninklijk Belgisch Instituut voor Natuurwetenschappen

In October 1960, the 26th Executive Board of the WHO resolved that postage stamps devoted to the Global Malaria Eradication Programme would be a valuable contribution to the dissemination of information and would stimulate interest in the battle against malaria [63]. It approved a plan for the issue of malaria eradication stamps and invited Member States to take part. It expressed its hope that Member States would find it possible to give the Organization either a percentage of the proceeds from the sale of such stamps, provide quantities of stamps for the WHO to sell internationally to philatelists, or make other suitable donations. The resolution was endorsed by the 14th WHA and the Universal Postal Union was invited to co- operate with the WHO and extend assistance and advice in the implementation of the proposed plan. [64] In May 1962 the Director General reported on progress at the 15th WHA, noting the number of collaborating countries issuing stamps and philatelic material [65]. The philanthropic character and equitable distribution of these anti-malaria issues was stressed (Fig. 8).

The stamps acted as advertisements and facilitated hemispheric good will, with success resting on universality of participation. International cooperation was facilitated with the assistance of the Pan American Health Organization and UNICEF, as well as non-malaria endemic donor countries [62]. A strict timeframe arose because issues produced after 31st December 1962 would not be considered part of WHO’s postage stamp initiative [65]. As the campaign emphasized the global importance of the malaria eradication effort, it was undertaken by more than 80 countries and included States with no indigenous malaria transmission (e.g., Lichtenstein). The temporary exclusion of sub-Saharan Africa was neither mentioned in the WHA resolutions that started the campaign, nor in those of following assemblies [22]. A partial or limited campaign took place in only three African countries, as from pilot projects it was not considered feasible to extend the program to the rest of the continent [66]. In a philatelic paradox, the first sub-Saharan African malaria stamps ever issued, which was on 6th April 1962, were as part of the campaign, and 30 African Nations made contributions (Table 2). While West African States issued stamps, East African countries used only special postmark cancellations (Kenya, Tanganyika, Uganda, Rhodesia, and Nyasaland). A few countries overprinted other non-malaria stamp issues (Guatemala, Mauritania, Mongolia). Two unauthorized overprinted stamps were produced from Sud Kasai, an unrecognized secessionist state within the Republic of the Congo.

**Table 2:** Countries issuing malaria stamps during first and second Global Eradication initiatives

Country	1962	1996-2020	Country	1962	1996-2020
Afghanistan	+	–	Maldive Islands	+	–
Albania	+	–	Mali	+	+
Argentina	+	–	Mauritania	+	–
Angola	+	+	Mexico	+	–
Bhutan^a^	+	–	Monaco	+	–
Bolivia	+	–	Mongolia	+	–
Brazil	+	–	Morocco	+	–
Bulgaria	+	–	Mozambique	+	+
Burundi	+	+	Nepal	+	–
Cambodia	+	–	Netherlands	-	+
Cape Verde	+	–	Nicaragua	+	–
Canal Zone Panama	+	–	Niger	+	+
Central African Republic	+	+	Nigeria	+	–
Ceylon	+	–	Panama	+	–
China	+	–	Papua New Guinea	+	–
Colombia	+	–	Paraguay	+	–
Comoros	+	–	Philippines	+	–
Congo, Brazzaville	+	–	Poland	+	–
Congo, Leopoldville	+	–	Portugal	+	–
Cuba	+	–	Portuguese India^d^	+	–
Cyprus	+	–	Ryukyu Islands	+	–
Czechoslovakia	+	–	Sao Tomé e Principe	+	+
Dahomey	+	–	Saudi Arabia	+	–
Dominican Republic	+	–	Senegal	+	–
Ethiopia	+	–	Sierra Leone	+	+
France	+	–	Solomon Islands	–	+
French Guinea^b^	–	+	Somalia	+	–
French Somaliland^c^	+	+	Spain	+	–
French Cameroun	+	–	Sudan	+	–
Gabon	+	–	Surinam	+	–
Ghana	+	+	Swaziland	+	–
Guinea	+	+	Switzerland	+	–
Guatemala	+	–	Syria	+	–
Haiti	+	–	Tanzania	–	+
Hungary	+	–	Tchad	+	+
India	+	+	Timor	+	–
Indonesia	+	–	Thailand	+	–
Iran	+	–	Togo	+	+
Iraq	+	–	Tunisia	+	–
Israel	+	–	Turkey	+	–
Italy	+	–	Uganda	–	+
Ivory Coast	+	–	United Arab Republic	+	–
Jordan	+	–	United Kingdom	–	+
Korea	+	–	Upper Volta^e^	+	+
Kuwait	+	–	UN Postal Administration	+	+
Laos	+	–	United States of America	+	–
Lebanon	+	–	USSR	+	–
Liberia	+	+	Vatican State	+	–
Libya	+	–	Venezuela	+	–
Liechtenstein	+	–	Vietnam	+	–
Macau	+	–	Yemen	+	–
Madagascar	+	–	Yugoslavia	+	–
Malaysia Federation	+	–			

^a^ Issue withdrawn in 1962 before official release

^b^ Now Republic of Guinea

^c^ Now Djibouti

^d^ Incorporated into India in 1961

^e^ Now Burkina Faso

**Fig. 9:**
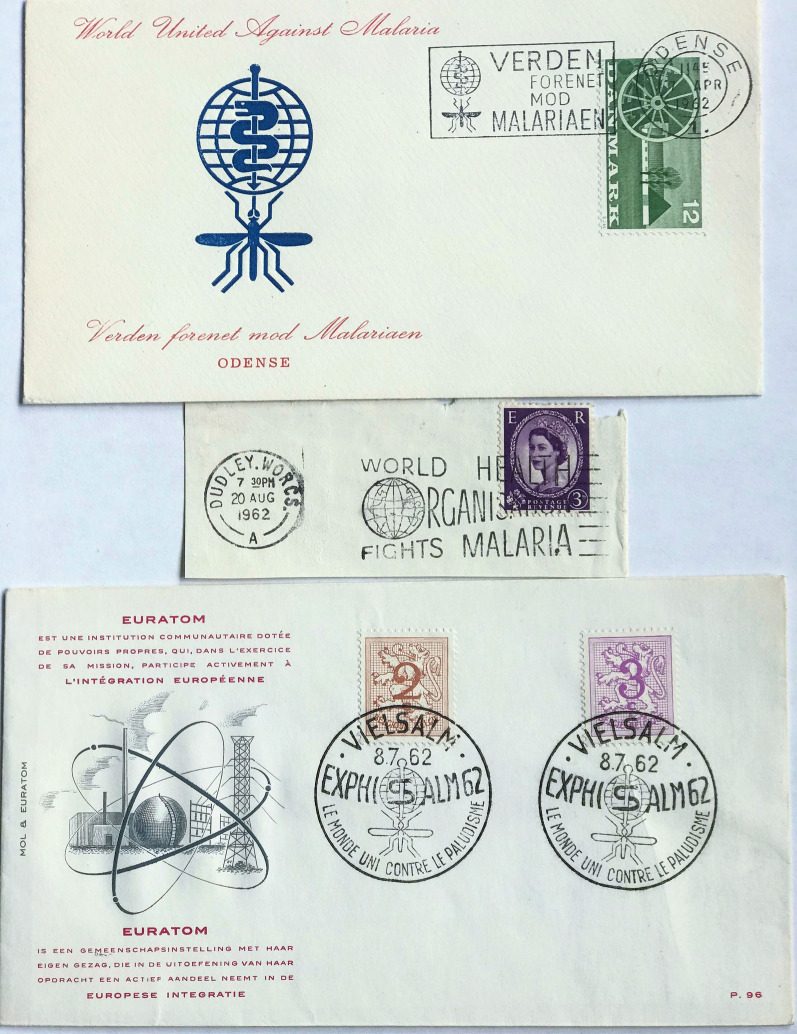
UK, Danish and Belgian slogan postmarks on the fight against malaria. Other countries using slogan postmarks in 1962 without issuing stamps were: Austria, British Guinea, Solomon Islands, Burma, Canada, Rhodesia and Nyasaland, Malta, New Zealand, Singapore, Zanzibar

Arrangements for the sale of donated stamps were made and communications maintained with partners and philatelic organizations, the stamp trade, and collectors. Publicity was increased by involving the International Boy Scouts Movement, chemical and pharmaceutical companies, together with mass information through radio and television. The WHO Headquarters printed visual material at its own expense and its sales agent, the Philatelic Agency for Malaria Eradication Postage Stamps, produced 720 short television films and 3180 phonographic records, which were distributed to radio and television stations for the ‘Stamp Out Malaria’ programme. Preparations were made for philatelic exhibitions in Geneva, Switzerland, and organized in Belgium, Germany, France, Korea, India, Iran, Philippines, Venezuela, Canada, UK, and several cities in the USA. In March 1962, the United Nations headquarters hosted a ceremony in New York marking the first day of issue of United Nations anti-malaria stamps. By the time of the 16th WHA in May 1963, 114 countries were involved, of which 98 postal administrations issued one or more malaria eradication postage stamps [67]. Some issued related philatelic material, such as souvenir sheets, first day covers, while 16 administrations printed special postmark cancellations (Fig. 9) [8]. Publicity was strengthened through press coverage and mass information media activities. The WHO Division of Public Information recorded press coverage by 30 countries, 1529 articles on the anti-malaria stamp campaign, the eradication programme, or the problem of malaria in general [67]. Many stamps carried the sign ‘World United Against Malaria’, and mail appeared with similar slogan postmarks. The UK postmark cancellation was applied to 200 million items. The quantity of stamps printed was enormous: USA 100 million, Vietnam 7 million, Niger 4.8 million, Czechoslovakia 4.5 million, Poland, 3.8 million, India 3.5 million, and Saudi Arabia 600,000 sets [67].

**Fig. 10:**
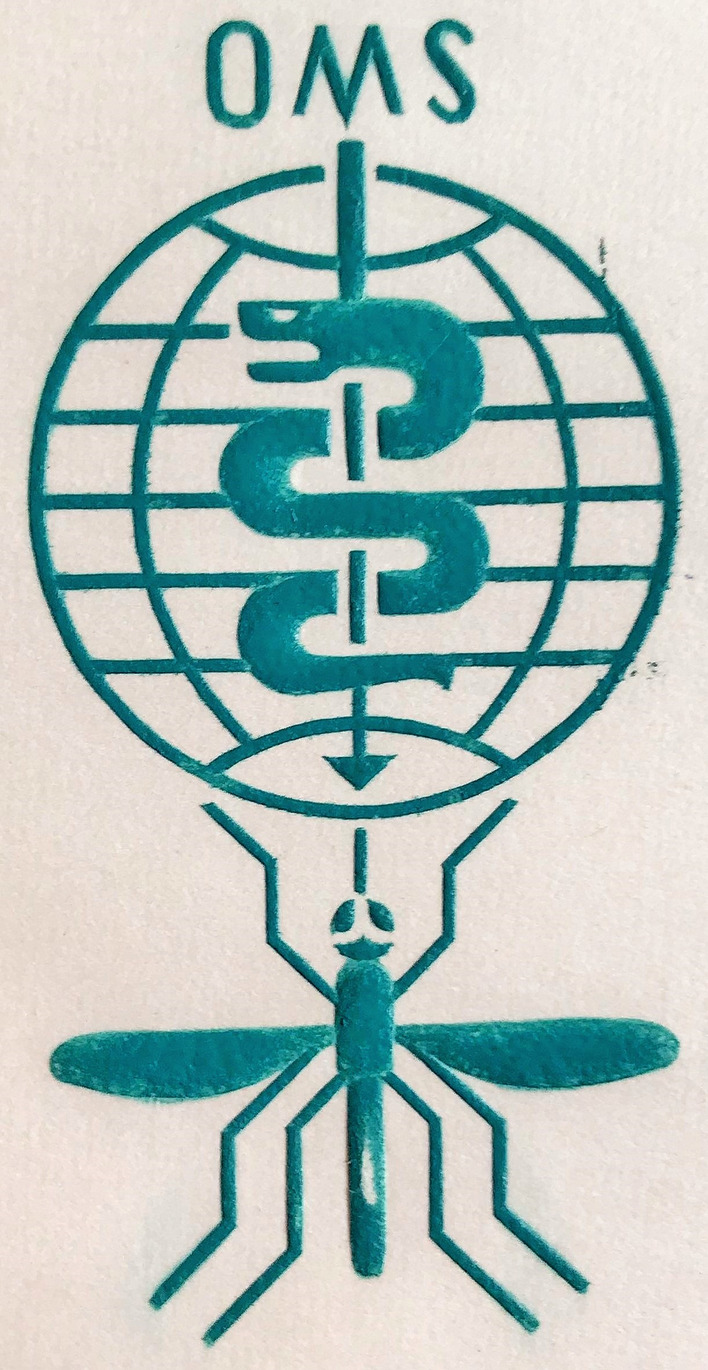
The WHO Global Malaria Eradication Campaign postage stamp emblem

Three alternative model stamp designs were made available to all WHO member States in 1961. Most nations closely following these suggested designs with many incorporating a mosquito supporting a globe and a caduceus symbol (Fig. 10), allowing typographical definition. West African countries widely used this design, and in the context of the presumed success of the programme, added a laurel corona surrounding the globe for embellishment (Fig. 8, Senegal). The Indian stamp which used this emblem represented what was probably the largest and most successful anti-malaria campaign in history. As shown in Fig. 10, to give emphasis to the importance of vector control, expressive power was reinforced by making the mosquito equivalent in size to the globe, suggesting that, despite its minute size, it challenged the greater whole on which it stood. Other designs, as well as including malaria symbols, embellished them-symbols of peace (Czechoslovakia); Red Cross emblems (Vietnam and East Germany); artistic and cultural representations of malaria (Lebanon); the USA showed its national symbol the eagle and Venezuela had an embossed globe. Saudi Arabian stamps included the Hegira year date (1381) and the Gregorian date (1962), showing the Islamic date to the left of the Gregorian date to the right. As Arabic reads from left to right this was inappropriate and fraudulent overprints were produced which were sent to the WHO with the dates reversed.

**Fig. 11:**
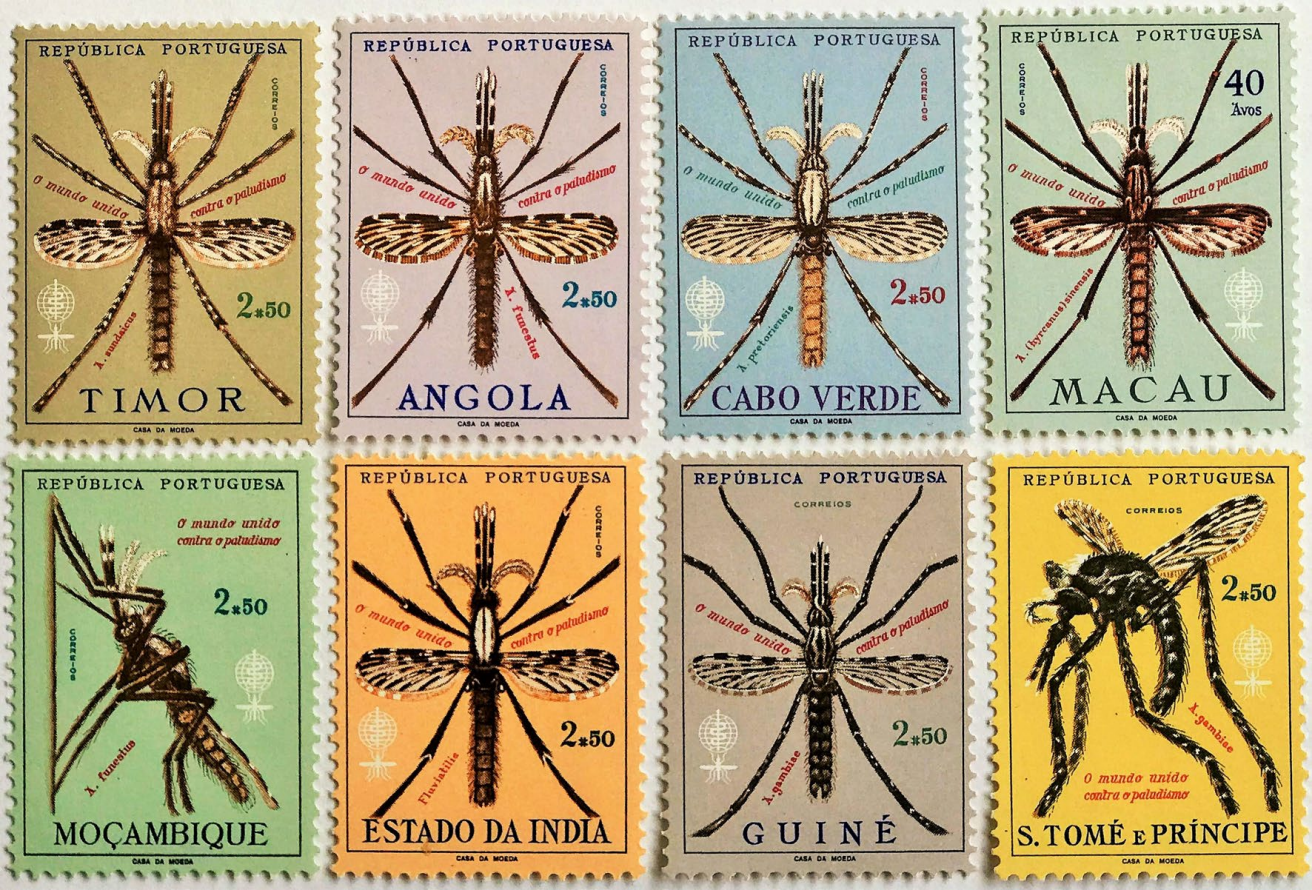
Anopheline species in Portuguese Territories in 1962 omnibus issue. Timor (*Anopheles sundaicus*), Angola (*A. funestus*), Cabo Verde (*A. pretoriensis*), Macau (*A. [hyrcanus] sinensis*), Mocambique (*A. funestus*), Estado da India (*A. fluviatilis*), Guiné (*A. gambiae*), S. Tomé e Principe (*A. gambiae*). The mosquito in the S.Tomé e Principe stamp is drawn in error with only four legs. Malaria stamps issued after 1962 have included other species: *A. stephensi* (Burundi, Mozambique, Guinée Bissau, Solomon Islands); *A. arabiensis* (Mozambique, Tchad); *A. maculipennis* and *A. atroparvus* (Sierra Leone)

Achieving design coherence across so many widely different cultures was challenging, because of an instinctive appeal to make use of culturally specific icons [15]. The problem could be held in check over the short period of one year, with the mosquito effectively accepted as the representative icon of the campaign. The *Anopheles* species, which illustrated the Portuguese Territories issues, were an outstanding example of use of this icon (Fig. 11).

**Fig. 12:**
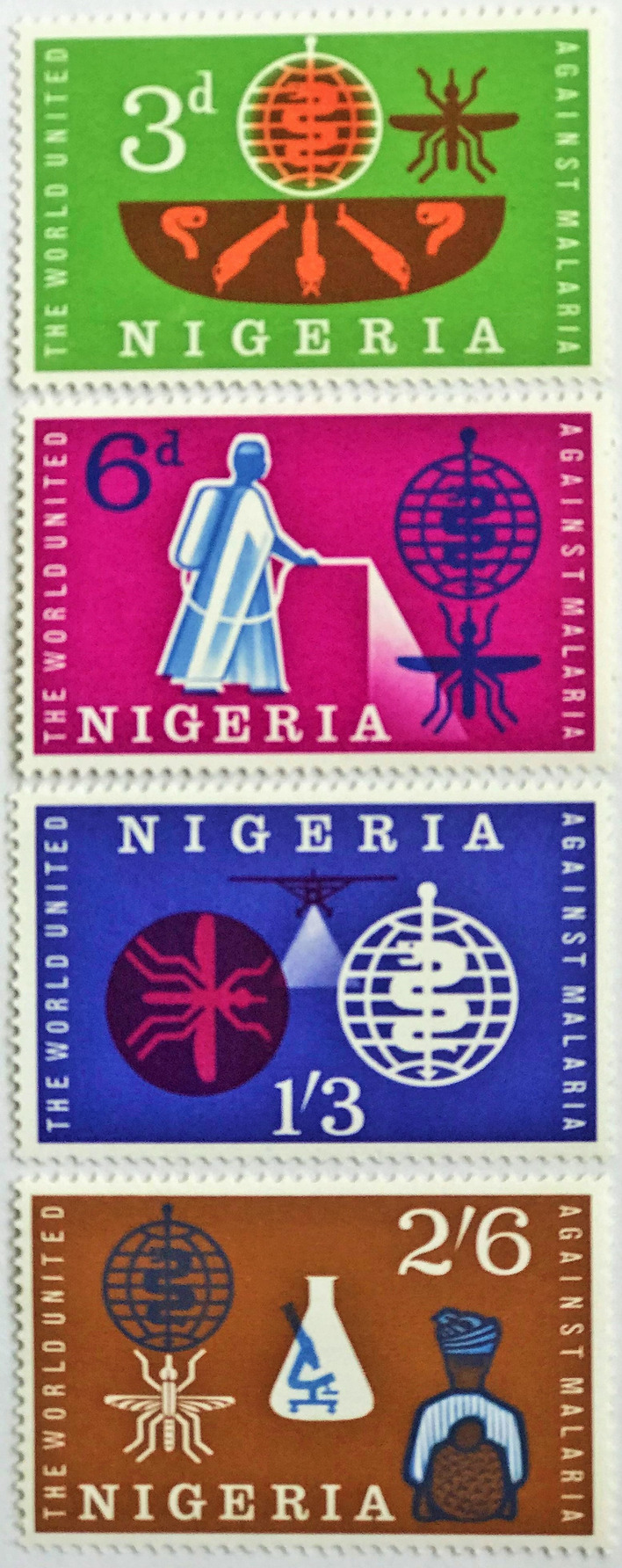
Nigerian issues for the 1962 Global Malaria Eradication Campaign. Scott commented on this specific stamp issue that ‘the role of typography in assuring legibility and visual impact is fundamental to its design and reflected in the choice of lettering, their integration of it into the overall design, and in the way the typography strengthens and enhances pictorial elements’ [15]. A typeface is selected that enhances the message of the issue with well-spaced modern typewriter typefaces

Some countries managed to shape the stamp’s imagery. The Nigerian anti-malaria issues retained the mosquito, but used text as an unfussy framing device, along with a capitalized text for the country name to establish a clear Nigerian identity (Fig. 12) [15]. Unfortunately, in this issue a design error showed the larva with a siphon, which meant it was not an *Anopheles* species. Similarly, the Polish issue had an error showing only white cells and no parasites in the microscopic image of a blood film (Fig. 8).

This huge ambitious philatelic contribution to the history of malaria control and eradication was not indexed, described, or mentioned in the standard reference textbook on malaria published in 1988 [68]. Was it later thought to be an irrelevance? Towards the end of the 1960 s the WHO campaign achieved malaria eradication in all developed countries where malaria was endemic, and interrupted transmission in most areas of tropical Asia and Latin America (e.g., in Brazil the number of cases decreased from 6 million to 37,000) [53]. These were extraordinary results, although in subsequent years the programme stalled. Vector insecticide resistance to DDT and the *Plasmodium* to chloroquine, heavily reduced the effectiveness of the eradication goal, to the point where malaria re-emerged in many areas. For the time being, the WHO abandoned the strategy of time-limited eradication and replaced it with that of the control, although eradication remained the goal, as reiterated at the 22nd WHA in 1969. The focus was now to be on reduction of morbidity and mortality [69]. The campaign cost an approximated 230 million dollars from 1955 to 1974. The final percentage contributed from postal revenue is unknown. The need to abandon the attempt was a major embarrassment to many governments and the eradication programme ceased to be mentioned [59]. Far fewer postal issues were released between 1965 and 1969. An exception was Gabon, which in 1966 produced an issue promoting malaria prophylaxis (Fig. 7). Iran in 1968 commemorated the 8th International Congress of Tropical Medicine and Malaria held in Tehran, and Nigeria and Cambodia commemorated the 20th Anniversary of the WHO, all themed around mosquitoes, although the Nigerian stamp used text promoting ‘inoculation against malaria’. In 1969 Jamaica, which was certified free of malaria in 1966, issued a single stamp from a set of three, without reference to its newly certified malaria-free status (Fig. 7).

### From 1970 to 1995 and the shift from malaria eradication to malaria control

**Fig. 13:**
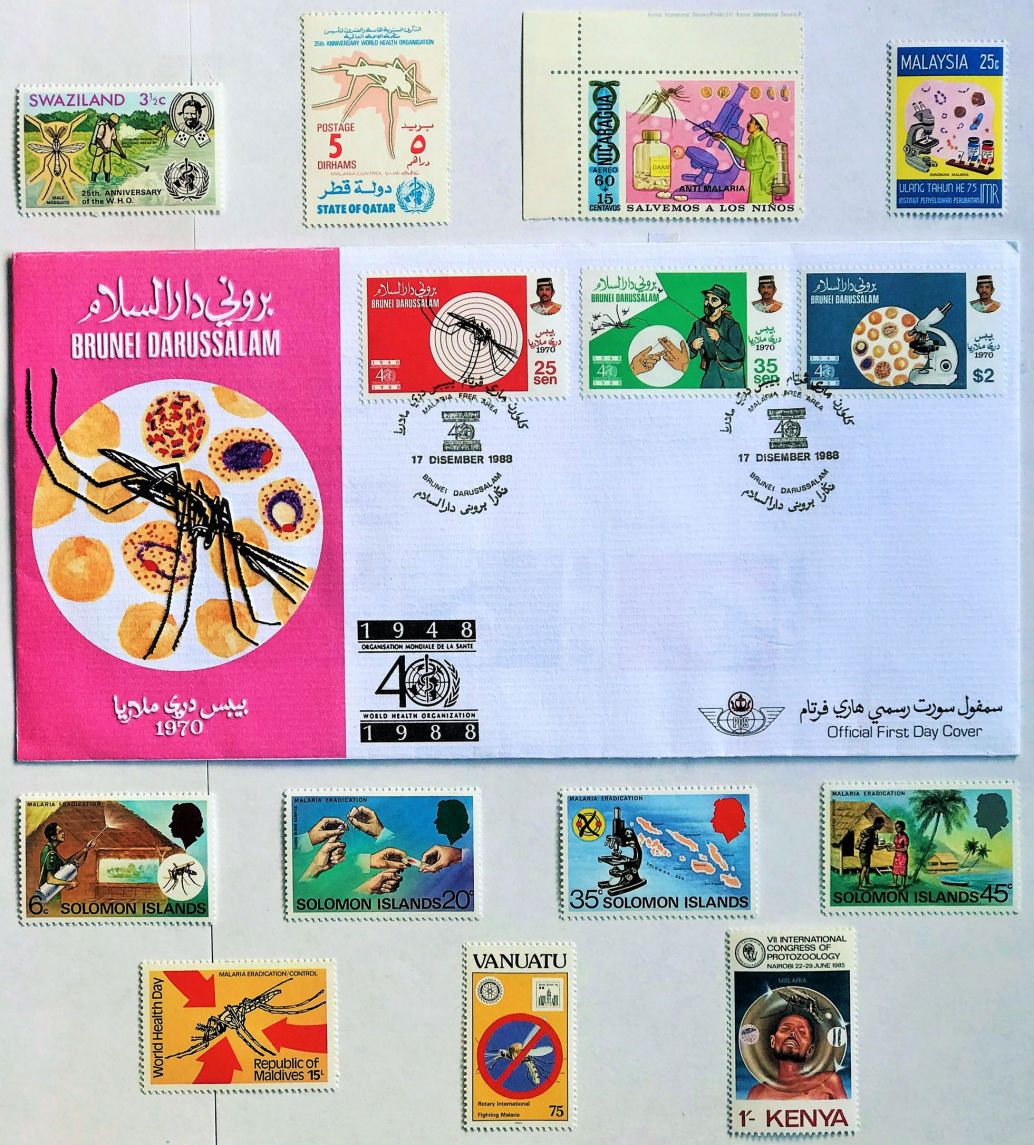
Stamps issued between 1970 and the Spirit of Dakar Call for Action in 1996. Issue dates: Swaziland, Qatar, and Nicaragua (1973); Malaysia (1976); Solomon Islands (1977); Kenya (1985); Brunei (1988); Maldives (1980); Vanuatu (1994). Not illustrated are the issues from Poland and Guyana in 1978 and Laos in 1988 and 1992

In 1975, the WHO European region (excluding Turkey) was considered malaria-free but from 1973 to 1978 a resurgence of endemic malaria occurred in in Central and South America, and notably in South-East Asia. Despite achievements by the global programme, a large reservoir of endemic malaria remained over most of the tropics. Reasons for failure were discussed in the Manson Oration by Bruce Chwatt in 1979 [70]. During the 1970 and 1980s international support for malaria control had declined. Stamps issued in the years between closure of the Global Malaria Eradication Campaign (1969) and the Spirit of Dakar Call for Action (1996) can be seen in Fig. 13.

As shown in Table 3 only twelve countries released issues during this quarter century, compared to 110 countries during the preceding 14 years of the Global Eradication Campaign. Annual incidence for country releases declined from a mean of eight per year, to one every three years, with only one from Europe. The 25th Anniversary of the WHO foundation in 1973 was commemorated in two issues from Swaziland and Qatar both showing mosquitoes and the 40th Anniversary by Laos in 1988 showing insecticide spraying. The Swaziland set was unusual in showing a child receiving malaria vaccination and an image of a male *Anopheles*. The declaration by the WHO that Brunei Darussalam had succeeded in eliminating malaria (achieved in 1970 with malaria transmission no longer occurring in this specific geographic area, certified in 1987) was also commemorated on the 40th anniversary of the WHO. This was the only country achieving elimination that subsequently issued a malaria related stamp, other than Jamaica in 1969 (Fig. 13).

**Table 3:** Number of countries issuing stamps related to malaria by WHO region and historical period^a^

WHO Region	1932–1954	1955–1969	1970–1995	1996–2020
Africa	1	35	2	18^b^
Eastern Mediterranean	0	15	1	0
Europe	1	20	1	2
Americas^c^	3	17	2	0
South-East Asia	0	21	4	1
Western Pacific	0	2	2	1
United Nations^d^	0	1	0	1
All regions	5	111	12	23

^a^ Excluded are States with botanical issues showing only Cinchona species, or insect transmission without stating malaria

^b^ Several of these African Nation’s postal administrations issued malaria-related stamps annually from 2012

^c^ Includes Caribbean region

^d^ The United Nations autonomously issue postally valid stamps from the UN agencies in Geneva, Vienna, and New York

In the late 1970 s WHO decided to incorporate anti-malaria work with the promotion of primary health care. This shift in strategy was captured in a series of health education stamps from Nicaragua released in 1973, with one dedicated to malaria showing a Daraprim (pyrimethamine) bottle and with text on the reverse side of the stamp on use of DDT and Daraprim prophylaxis (Fig. 13) [71]. During the 1980 s two further issues were released, from the Maldives in 1988 to commemorate World Heath Day, and from Kenya during the 7th International Congress of Parasitology held in Nairobi (Fig. 13). The 4th International Congress of Parasitologists (Warsaw) in 1978 showed *Anopheles* and red cells infected by a malaria parasite, in a commemorative Polish release, and Laos insecticide spraying in issues in 1988 and 1992. In 1992, the WHO drew up a new strategy with emphasis on early diagnosis and immediate treatment in the context of programmes managed by the basic health care system. Many countries, such as Thailand, China, Brazil, Solomon Islands, Philippines, Vietnam, obtained good results in terms of control. For many others, and especially for those in sub-Saharan Africa, the malaria situation was still critical. Malaria control became a pivotal issue in the late 1990 s during election of a new WHO Director General [72].

### From 1996 to 2020 during the Roll Back Malaria Initiative and the second global campaign

The Global Malaria Control and Elimination Initiatives of 1996–2020 are outlined in Table 4. There were few malaria-related stamp releases in this period before 2010. Early Mali stamps on the management of severe malaria (2000), and issues from Uganda, Tanzania (2003) and Liberia (2006), highlighted Millenium Development Goal Six to combat HIV/AIDS, malaria, and other diseases. Malaria management and control were integral to reductions in child mortality and to achieve this the international community aimed to scale up control measures. A new eradication strategy was developed based on: (1) aggressive control in highly endemic countries, (2) progressive elimination from the endemic margins, to shrink the malaria map; and (3) research into vaccines, drugs, diagnostics, insecticides, genetic vector studies, and delivery methods that could reach at-risk populations [73, 74]. The WHA in 2015 adopted a Global Technical Strategy of achieving a malaria-free world, including a goal of at least 35 countries eliminating malaria by 2030 [74]. The agenda addressed resistance of *Plasmodium falciparum* to anti-malarial drugs, resistance of *Anopheles* to insecticides, asymptomatic malaria infections, and community level involvement. By the end of 2020, 24 countries had reported interrupting malaria transmission for three years or more. Of these, 11 were certified malaria-free by the WHO [75].

**Table 4:** Global Malaria Control and Elimination Initiatives 1996–2020

Initiative	Acronym	Start year	Orientation	References
Spirit of Dakar Call for Action	–	1996	Hypotheses concerning new initiative to control malaria across Africa proposed by WHO Africa Regional Office and World Bank	[24]
Multilateral Initiative on Malaria	MIM	1997	International alliance on scientific research in Africa by capacity building, collaboration, and coordination; notably long-lasting insecticide treated nets; rapid diagnostic tests; artemisinin-based therapies	[76]
Roll Back Malaria Partnership	RBM	1998	Global Action Plan of WHO, World Bank, UN Development Program, UNICEF Abuja Declaration in 2000 to halve malaria mortality by 2010. In 2020 offers collaborative platform for malaria of over 500 organizations	[77]
Medicines for Malaria Venture	MMV	1999	Public-private partnership for discovering, developing, and delivering new drugs	[78]
Global Fund to fight AIDS, TB, and Malaria	Global Fund	2002	Financial organization supporting prevention and treatment programmes to end epidemics, to achieve the UN Sustainable Development Goals	[79]
United States Presidents Malaria Initiative	PMI	2005	Country-managed prevention, care, and treatment in 24 high burden countries in sub-Saharan Africa and 3 countries in S.E. Asia Greater Mekong sub-region	[80]
Bill and Melinda Gates Foundation Forum	BMG	2007	Call for renewed commitment to eradicate malaria	[81]
Malaria Elimination Initiative	MEI	2007	Multidisciplinary team, University of California, San Francisco, advancing policy and practice	[82]
Malaria Eradication Research Agenda	malERA	2008	Scientific consultative process on vaccines, drugs, diagnostics, insecticides, interventions, and delivery methods for at-risk populations	[83]
Malaria Eradication Scientific Alliance	MESA	2008	Barcelona Institute of Global Health Strategic alliance advancing the science of malaria eradication	[84]
Asia-Pacific Malaria Elimination Network^a^	APMEN	2009	18 Asia Pacific countries that are pursuing elimination	[85]
Action and Investment to Defeat Malaria	AIM	2015	Advocacy plan developed by RBM partnership to unite global community for achieving the 2030 goals	[86]
WHO Global Technical Strategy for Malaria	GTS	2016	Technical framework for control and elimination between 2016–2030. 35 countries met malaria eliminating criteria in 2015	[87]
Lancet Commission on Malaria Eradication	–	2016	Development of roadmap for malaria eradication within a generation.	[25]
Strategic Advisory Group on Malaria Eradication	SAGme	2016	WHO convened group to determine feasibility of malaria eradication on basis of current trends	[88]
WHO Malaria Elimination Certification Panel^b^	MECP	2017	Recommends whether country malaria elimination can be certified	[89]
WHO E-2020 Initiative	E-2020	2017	Support for 21 countries to get zero malaria cases by 2020 timeline	[90]
WHO Malaria Elimination Oversight Committee	MEOC	2018	Overview of country/regional progress to elimination with focus on 21 countries	[91]
High Burden to High Impact approach	HBHI	2018	WHO and RBM initiative with focus on 11 highest burden countries (10 in Africa) to achieve GTS targets for 2025 through country-owned initiatives	[92]
Malaria Vaccine Implementation Programme	MVIP	2019	Implementation of first malaria vaccine in Africa in child immunization	[93]

^a^ Other regional elimination networks include: EMMIE (Elimination of Malaria in Mesoamerica); Malaria Zero (Dominican Republic, Haiti); APLMA (Asia Pacific Leaders Malaria Alliance); Malaria Elimination in the Greater Mekong (Cambodia, Laos, Myanmar, Thailand, Vietnam, Yunnan Province, China); Malaria-Free Arabian Peninsula (Bahrain, Kuwait, Oman, Qatar, Saudi Arabia, United Arab Emirates, Yemen); WHO-EURO (Armenia, Azerbaijan, Georgia, Kazakhstan, Krgyzstan, Tajikistan, Turkey, Turkmenistan, Uzbekistan); Elimination 8 (Botswana, Namibia, South Africa, Swaziland, Angola, Mozambique, Zambia, Zimbabwe); African Leaders Malaria Alliance (ALMA) [88]

^b^ Since the early 1960 s the WHO has maintained an official register of areas where malaria elimination has been achieved

**Fig. 14:**
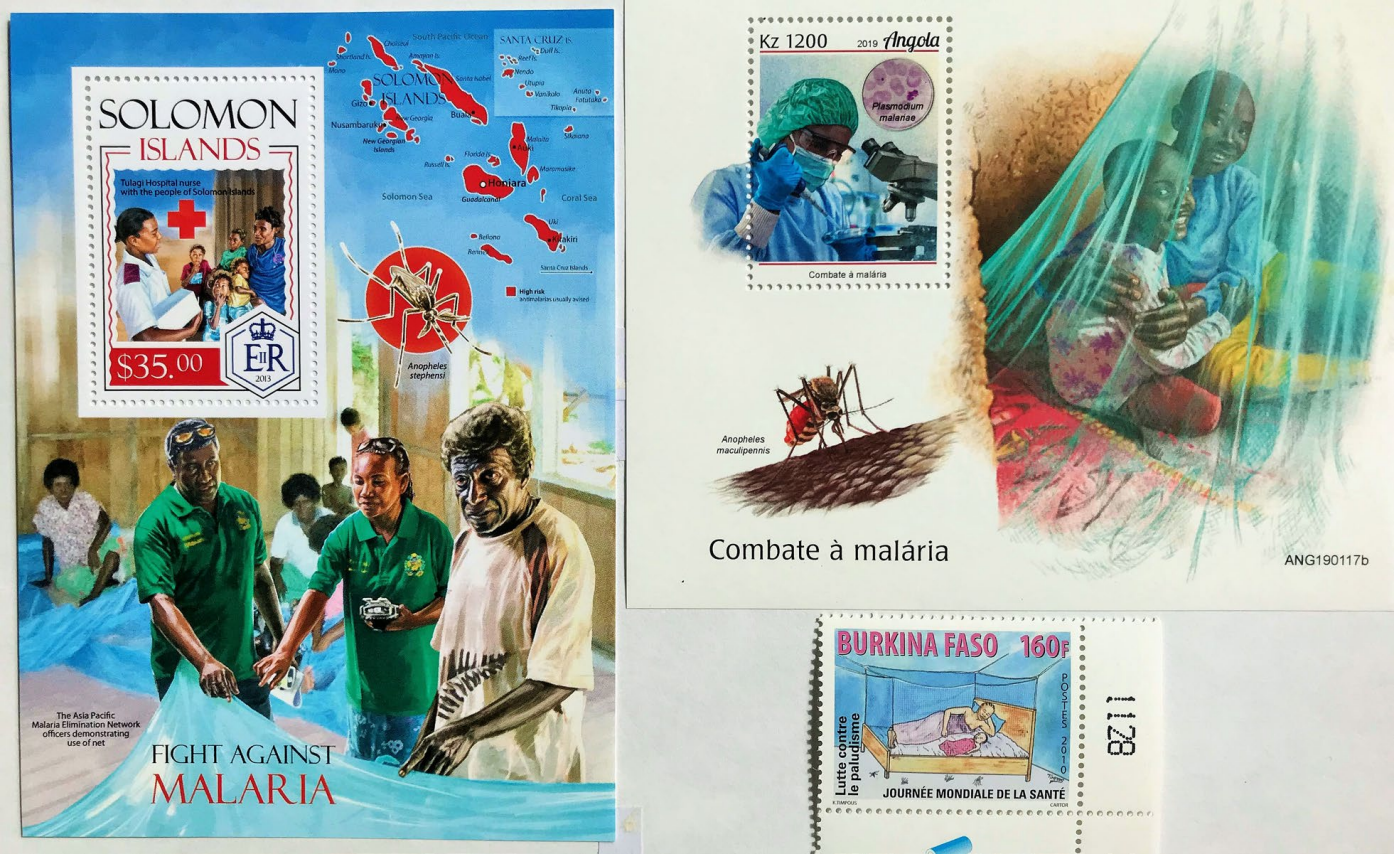
Contrasting stamp designs illustrating bed net use in early 21st century issues. Burkina Faso (2010); Solomon Islands (2013), Angola (2019). The Solomon Islands issue offers a picture of the Asian Pacific Malaria Elimination Network (APMEN, Table 4) demonstrating use of a net

Control initiatives were represented across a wide range of philatelic issues mostly from African states. Many were aimed principally at the stamp collector, and unlike the 1962 omnibus issue, there was little emphasis on the WHO. Some of the smallest countries produced the largest number of stamps, which were often extravagant in colour, form, and imagery. Although some issues clearly and simply outline surveillance and diagnostic methods, latterly, sophisticated photomontage techniques have been used to contemporize images. Differences can be observed when comparing illustration of insecticide bed net issue in the issue from Burkina Faso (2010), with those of the Solomon Islands (2013) and Angola (2019) (Fig. 14). In the Angolan and Solomon Island miniature sheet images, the challenging expressions of the workers and children appeal to the observer to utilize bed nets. In contrast the Burkina Faso stamp is almost simplistic, but homely, which could appeal in more rural settings. The Angolan stamp highlights *Plasmodium malariae* which is a neglected species in Africa. The size of the Solomon Islands miniature sheet, the range of iconographic images and the inclusion of text stating the sheet’s purpose to demonstrate use of bed nets, creates the impression of a poster.

**Fig. 15:**
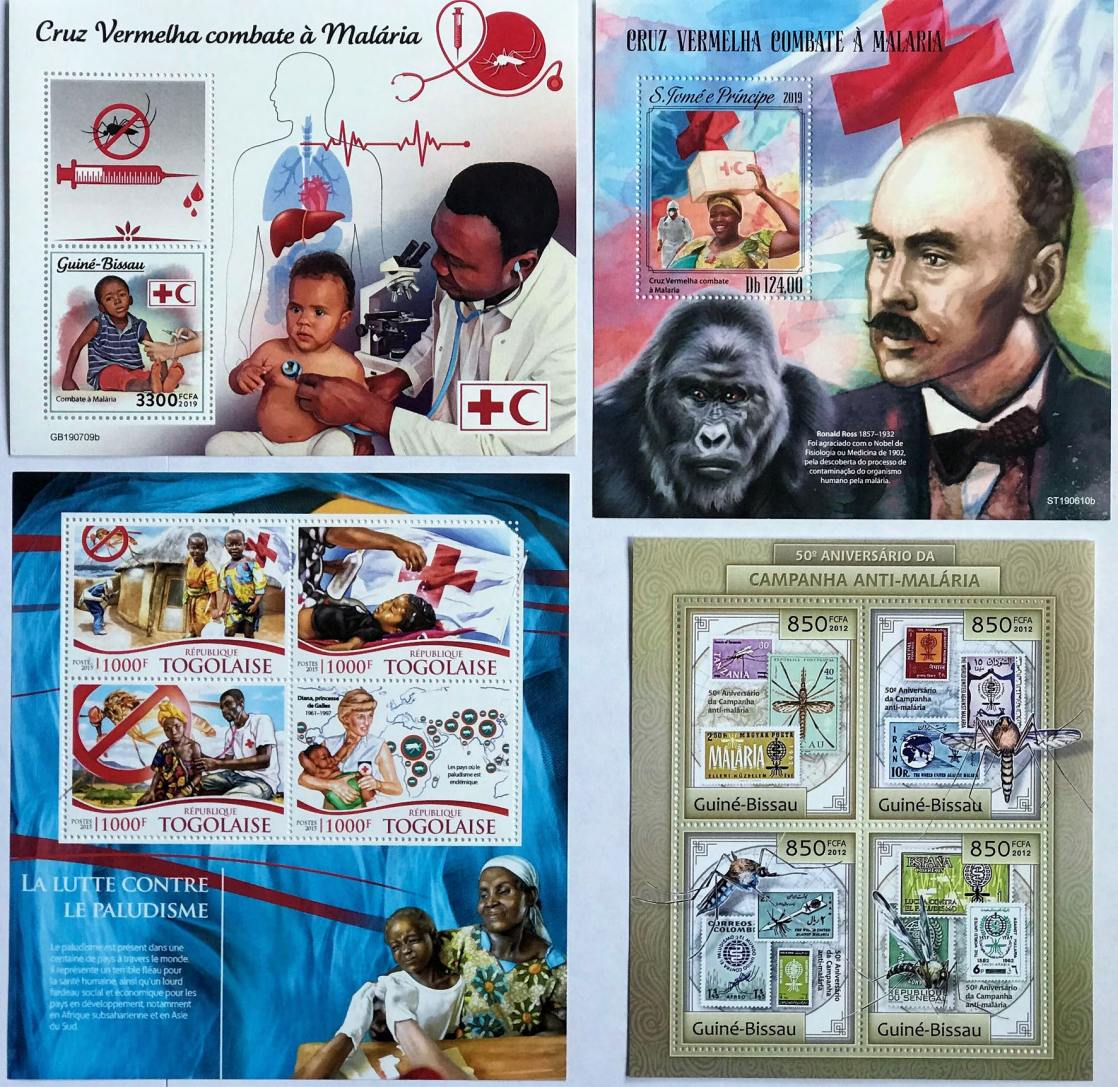
Examples of West African stamps issued following the Roll Back Malaria Partnership. Guiné-Bissau (2019 and 2012); S.Tomé e Principe (2019); Togo (2015)

Of twenty-three countries issuing malaria-related stamps since 1996 (Table 3), many issued miniature sheets with a montage of figurative images. The issue from Guinée-Bissau presented a palimpsest of the 1962 anti-malaria campaign stamps within the later 2012 stamp issue, which marked the 50th anniversary of the campaign, and served a specific indexical function [94]. Some African countries produced annual malaria issues between 2015 and 2020, a regularity that had not occurred after the omnibus issues. Also different was that the earlier Global Malaria Eradication Campaign omnibus issues favoured a single motif and an absence of human images. While visually attractive, later use of multiple images within the same stamp may have undermined the clear message of a single key iconic image. A good example is the 2019 miniature sheet from S. Tomé e Principe, which shows a non-human primate (implying zoonotic transmission with simian malaria), Red Cross symbols and Ronald Ross, making for a confusing and mixed take-home message (Fig. 15). This is also demonstrated in the Guinée-Bissau issue of the same year, which has a montage of pictures of syringes, indicating either venepuncture, vaccination, or parenteral injection, with images of the Red Cross and Crescent, a healthy-looking child, an electrocardiograph, microscope, cardiac and hepatic anatomy, and a mosquito (Fig. 15).

Modern productions are specialized philatelic items with decorative margins and boldness of design, and although designed mainly for collectors, have normal postal validity. Designers have used considerable ingenuity, such as devising issues showing a stamp within a stamp, to provide continuity with the 1962 omnibus issues. The stamp in Fig. 15 from Togo shows Princess Diana (1961–1997) alongside a global map of endemic malaria. These stamps are likely to have limited postal use in the home country. Possibly some go straight from the printing presses of London, New York, and South Africa to collectors, and may not be easily available through the post offices of the countries that issued them. They are aimed primarily at first world collectors and provide a source of Government revenue, with a tendency for numerous high value stamps. Images picked up from the media, science or other cultural sources are used, and can offer authentic information about malaria in their country. These stamps are documents of enduring fascination and significance.

**Fig. 16:**
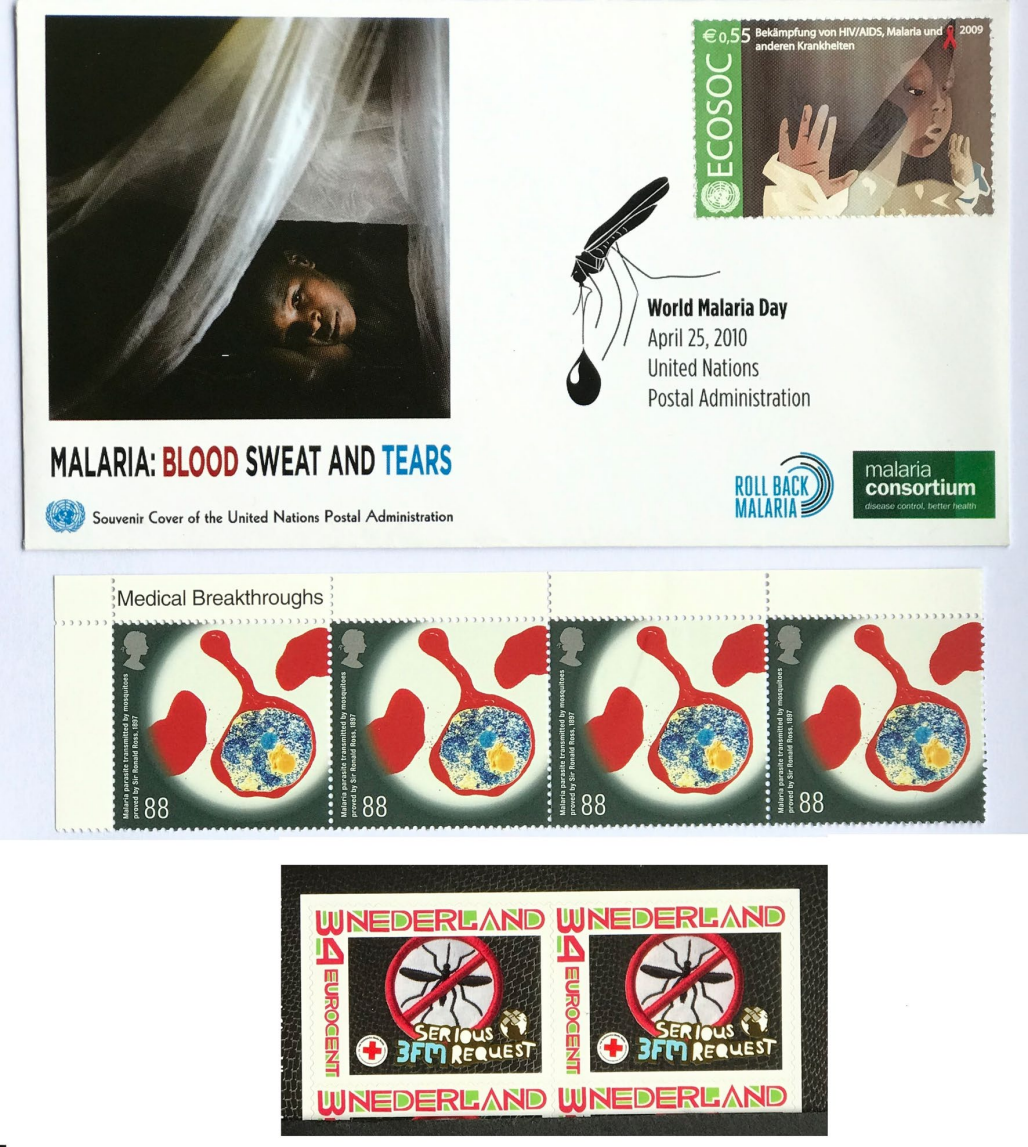
Twenty-first century European issues. United Nations World Malaria Day cover April 2010, with permission United Nations Postal Administration, Europe. United Kingdom Medical Breakthroughs issue showing a colour transmission electron micrograph of a red cell infected with a malaria parasite, with permission UK Royal Mail (Stamp design © Royal Mail Group Limited). The Netherlands ‘Stop Malaria’ stamp was released as a multimedia fundraising event for the International Red Cross before New Year 2010 and was valid for a six-week period

Two contrasting issues were forthcoming from Europe during this period. In 2009 a stamp of the United Nations Postal Administration was released on a First Day Cover for World Malaria Day promoting the Economic and Social Council (ECOSEC), which co-ordinates the work of 14 UN agencies, although the stamp highlighted HIV, AIDS, and malaria recognizing the interplay between malaria and other infections. The cover was titled ‘Malaria: Blood, Sweat and Tears’ (Fig. 16). In contrast the UK, in the same year, issued a stamp celebrating medical breakthroughs of a colour transmission electron micrograph of a red cell infected by a malaria parasite, which had small font text stating mosquito transmission of malaria was proved by Ross in 1897 (Fig. 16). This more academic/scientific approach as captured in this recent UK issue differs from the ‘Stop Malaria’ stamp released in the same year as part of a Dutch multimedia fundraising event for the International Red Cross hosted by radio stations in the weeks before New Year 2010.

## Discussion

Over the decades, malaria postage stamps have helped to establish, not simply awareness, but commitment to malaria control. Malaria stamps have reached an international audience, using familiar icons, embellished with cultural signs and symbols. This success was a substantial accomplishment that had no forerunner. The role of the WHO was critical as it undertook technical co-ordination of national responses and endeavoured to harness a global philatelic initiative in the promotion of its goal for eradication. In the beginning, the 1962 WHO-endorsed stamp issues were a unique philatelic event on a grandiose scale, echoing the spirit that ‘we are all in this together.’ It was a call to countries to get involved. Between 1948 and 1982, the incidence of WHO-related stamps issued by Member States, other States, and the United Nations was considerable [8]. Stamps also raised funds although this would have been a relatively small financial contribution to the overall costs, and the final amount of the philatelic component is uncertain. In present day terms, it would be negligible in the context of the US$ 6.6 billion per annum needed by 2020 to achieve global malaria targets by 2030 [95].

Stamps have been studied very little as a tool to communicate health awareness although their use is assumed to be beneficial. There is only limited evidence they are an educational and knowledge resource, although they may act as a vehicle for philanthropic contributions [96]. It is difficult to judge the impact of stamps in promoting health awareness within countries, as issues are generally available for only a limited period, and it is questionable how many of these penetrate the poorest rural areas, where electronic forms of communication have increased rapidly. Yet communities need health awareness programmes [97], especially as malaria goals have changed and stamps have not always given accurate information. That mass communication strategies, including postage stamps, could be effective in delivering general health messages was suggested in the 1970s by David Morley and Maurice King [71, 98]. As postage stamps index World Health Days, they can reference a variety of interventions and newly emerging public health problems [99]. An example of this was the recent issue by the UN and 21 countries to honour healthcare workers and patients during the COVID-19 pandemic [10].

Postage stamps played a minor role in the WHO Smallpox Eradication Programme, which commenced in 1966 [100], but for the WHO Polio Eradication Programme, (1988-onwards), many issues were released on immunization. Immunization is a relatively simple message to convey and is a single intervention for a single virus, in contrast to the multiple messages required for malaria eradication [101]. Malaria is not a specific pathogen, as it comprises seven distinct species infecting humans, with *P. falciparum* and *Plasmodium vivax* as the focus of eradication efforts. While it has been convenient to use the term ‘malaria eradication’ for funding and advocacy purposes, this is technically incorrect [102]. The term ‘elimination’ is used when malaria transmission is no longer occurring in a specific geographic area, whereas ‘eradication’ describes world-wide zero incidence of a specific pathogen due to deliberate efforts with no risk of re-introduction. The messaging on stamps mostly does not correspond with these definitions. Very few national postal administrations have issued stamps commemorating malaria elimination.

Recent malaria issues that have focused on bed nets could increase their acceptance and use (Fig. 14). Other recent philatelic themes have included spraying with insecticides and diagnostics, but intermittent preventive treatment with anti-malarials, malaria control in pregnancy, asymptomatic malaria, or malaria vaccination have not yet been addressed. It may be argued that the public pays only passing attention to stamp design and messages, but they do represent a form of mass-produced art, seen daily or frequently by many citizens. To raise health awareness requires wide stamp circulation, low face value for use, and an attractive design. They must be accurate, with focused themes that avoid multiple messaging, highlight priority interventions, and maintain thematic continuity between sequential annual issues.

Today more than 90 countries remain endemic for malaria and none from sub-Saharan Africa have received a WHO Certification of Elimination (three consecutive years of zero indigenous cases). In 2018, around 68 % of the estimated case burden and 65% of the estimated deaths globally occurred in 10 countries in sub-Saharan Africa and in India. Nigeria accounted for the highest proportion of cases globally (25%) [101]. The current Global Technical Strategy to support a High Burden to High Impact approach focuses on the 11 highest burden countries (Nigeria, Democratic Republic of the Congo, Mozambique, India, Uganda, Burkina Faso, Ghana, Niger, Cameroon, Mali, United Republic of Tanzania) to achieve the 2025 targets to reduce malaria cases and deaths by at least 75 % through country owned initiatives (Table 4). A number of these countries have not used philatelic products during the current initiative, in particular Nigeria, which has the highest malaria burden (25%) and last issued a malaria-related postage stamp in 1968.

In conclusion, stamps can make an impact. Some stamp designs have an established provenance, and some stamp icons are well known, even by non-collectors. These include the Maltese Cross on the world’s first stamp, an emblem of the Knights Hospitaller, who established the great hospitals during the time of the crusades from western Europe to the Levant, and a symbol that is shown on the 1840 ‘Penny Black’ [26]. In 2022, as 60 years is approached since the first WHO co-ordinated omnibus issue, perhaps an official rallying cry of encouragement could again draw on the aid of the humble postage stamp.

## Data Availability

Not applicable.

## Abbreviations

DDT: Dichlorodiphenyltrichloroethane
WHO: World Health Organization
UNICEF: United Nations International Children’s Emergency Fund
WHA: World Health Assembly
UN: United Nations
UK: United Kingdom
HIV: Human immunodeficiency virus
GTS: WHO Global Technical Strategy for Malaria
RBM: Roll Back Malaria
